# Inhibition of histone methyltransferase EZH2 for immune interception of colorectal cancer in Lynch syndrome

**DOI:** 10.1172/jci.insight.177545

**Published:** 2025-02-13

**Authors:** Charles M. Bowen, Fahriye Duzagac, Abel Martel-Martel, Laura Reyes-Uribe, Mahira Zaheer, Jacklyn Thompson, Nan Deng, Ria Sinha, Soham Mazumdar, Melissa W. Taggart, Abhinav K. Jain, Elena Tosti, Winfried Edelmann, Krishna M. Sinha, Eduardo Vilar

**Affiliations:** 1 Department of Clinical Cancer Prevention,; 2 Department of Pathology, and; 3 Department of Epigenetics and Molecular Carcinogenesis, The University of Texas MD Anderson Cancer Center, Houston, Texas, USA.; 4 Department of Cell Biology, Albert Einstein College of Medicine, Bronx, New York, USA.

**Keywords:** Immunology, Oncology, Colorectal cancer, Epigenetics, Immunotherapy

## Abstract

Colorectal precancers in Lynch syndrome (LS) exhibit a distinct immune profile, presenting unique opportunities for developing immune-interception strategies to prevent carcinogenesis. Epigenetic modulation by EZH2 of immune-related genes is implicated in the carcinogenesis of different cancer types, including colorectal cancer. This study utilizes a mouse model of LS and ex vivo colonic organoids to assess the effects of the EZH2 inhibitor GSK503 on immune regulatory pathways, tumorigenesis, and epigenetic reprogramming. Our findings revealed that GSK503 significantly increased CD4^+^ and CD8^+^ T cells in both splenocytes and colonic mucosa of treated mice compared with controls. Additionally, a preventive dose of GSK503 over 9 weeks notably reduced adenoma multiplicity, demonstrating its efficacy as a preventive modality. Single-cell RNA-Seq and molecular analyses showed activation of immune and apoptotic markers, along with a reduction in H3K27 methylation levels in colonic crypts. ChIP sequencing further revealed decreased levels of H3K27me3 and H3K4me1, while levels of the active enhancer marks H3K4me3 and H3K27Ac increased in treated mice. Collectively, these findings indicate that EZH2 inhibition enhances immune responses through epigenetic reprogramming in the genome of LS mice, establishing a promising framework for the clinical development of EZH2 inhibitors as a cancer prevention strategy for LS carriers.

## Abstract 2

EZH2 inhibition modulates the immune landscape in a mouse model of Lynch Syndrome colorectal cancer with promising insight for launching a phase 1 trial.

## Graphical abstract

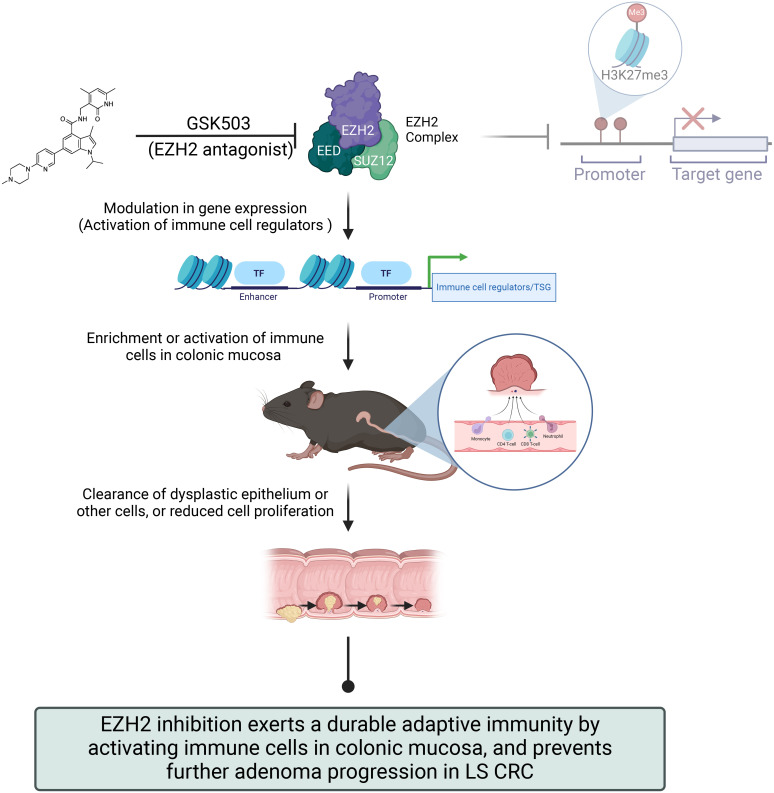

## Introduction

Colorectal cancer (CRC) remains the third leading cause of cancer-related deaths, affecting both men and women (1). The lifetime risk of developing CRC in the general population is nearly 5 percent, while individuals with hereditary cancer syndromes face a 10- to 20-fold higher risk (2). Lynch syndrome (LS; OMIM# 120435), the most common cause of hereditary CRC, has an autosomal dominant inheritance pattern and accounts for 2%–4% of CRC cases. LS arises from heterozygous germline mutations in 1 of 4 DNA mismatch repair (MMR) genes (*MLH1*, *MSH2*, *MSH6*, and *PMS2*) responsible for postreplicative DNA proofreading to ensure genomic integrity (3). LS-associated CRC occurs when normal colorectal cells become MMR deficient (MMRd) due to a somatic hit in the alternative allele of the MMR gene carrying the germline mutations (4). This inactivation leads to the accumulation of numerous base-to-base mismatches and insertion-deletion mutations (indels) in oncogenes and tumor suppressor genes, which are critical for maintaining normal tissue dynamics, thereby promoting carcinogenesis (5). In addition, MMRd can also result from sporadic loss of DNA MMR proteins (sMMRd). In fact, in specific sporadic CRC subtypes, such as the CpG island methylator phenotype (CIMP), global epigenetic modifications lead to extensive hypermethylation of promoter CpG islands, resulting in higher rates of gene methylation (6). CIMP^+^ CRCs exhibit epigenetic silencing of *MLH1*, which contributes to functional sMMRd (7).

Despite their shared loss of MMR functionality, LS-associated CRC and sMMRd CRC have marked differences, particularly in their immune microenvironments (8). Studies have shown that the immune microenvironment of LS-associated CRC has higher levels of recruited and infiltrating immune cells, especially cytotoxic CD8^+^ T cells. This is likely due to persistent neoantigen priming, a process referred to as “self-educating,” which enhances immune responses in patients with LS (8). Research indicates that the enrichment of cytotoxic T cell infiltration supports tumor immunity and therapeutic efficacy, leading to improved patient survival, partly through epigenetic regulation of Th1-type chemokine expression (9, 10).

Epigenetic alterations are critical in CRC initiation, promotion, and metastasis (7, 11, 12) and have been linked to the pathogenesis of LS CRC (13, 14). Previous studies suggest that these alterations facilitate the transformation of colonic epithelial cells, thus contributing to carcinogenesis through 3 main mechanisms: (a) DNA methylation patterns, (b) histone acetylation/deacetylation, and (c) destabilization of growth and transcription factor pathways (15). Histone modifications, regulated by deacetylase and methyltransferase enzymes, also play a role in immune homeostasis within the intestinal mucosa of inflammatory bowel disease and various CRC subtypes (9, 16). Although inflammatory bowel disease and LS CRC differ in etiology, immune dysregulation — such as immune evasion and suppression — has been recognized as a protumorigenic mechanism in LS CRC progression, potentially driven by epigenetic dysregulation within the tissue microenvironment (8, 10).

Enhancer of zeste homolog 2 (EZH2), a histone methyltransferase, contributes to immune regulation by trimethylating lysine 27 residues on histone H3 (17, 18). In several cancers, including lung, colon, breast, pancreas, and prostate, overexpression of EZH2 plays two key roles: (a) modulation of innate and adaptive immune pathways and (b) regulation of stem cell self-renewal, differentiation, and lineage fate (19–21). Previous studies from our group have shown that LS precancers and cancers possess a distinct immune profile and neoantigen repertoire (22–24). Targeting these characteristics may facilitate the development of vaccines or immune checkpoint inhibitors as preventive interventions for MMRd carcinogenesis. Building on these findings, we aimed to investigate the role of EZH2 inhibition as an immune-modulating strategy in LS colorectal mucosa using a murine model of LS colorectal carcinogenesis (herein referred to as *VCMsh2T*^Hu^) (25). To test this hypothesis, we utilized the selective small-molecule inhibitor of EZH2, GSK503, which has shown specific targeting of the SET methyltransferase domain of EZH2 with favorable potency and pharmacokinetics in mice (26, 27).

## Results

### Targeting EZH2 alters gene expression in dysplastic tissue and enhances immune cell abundance in a mouse-derived model of LS.

**Figure 1:**
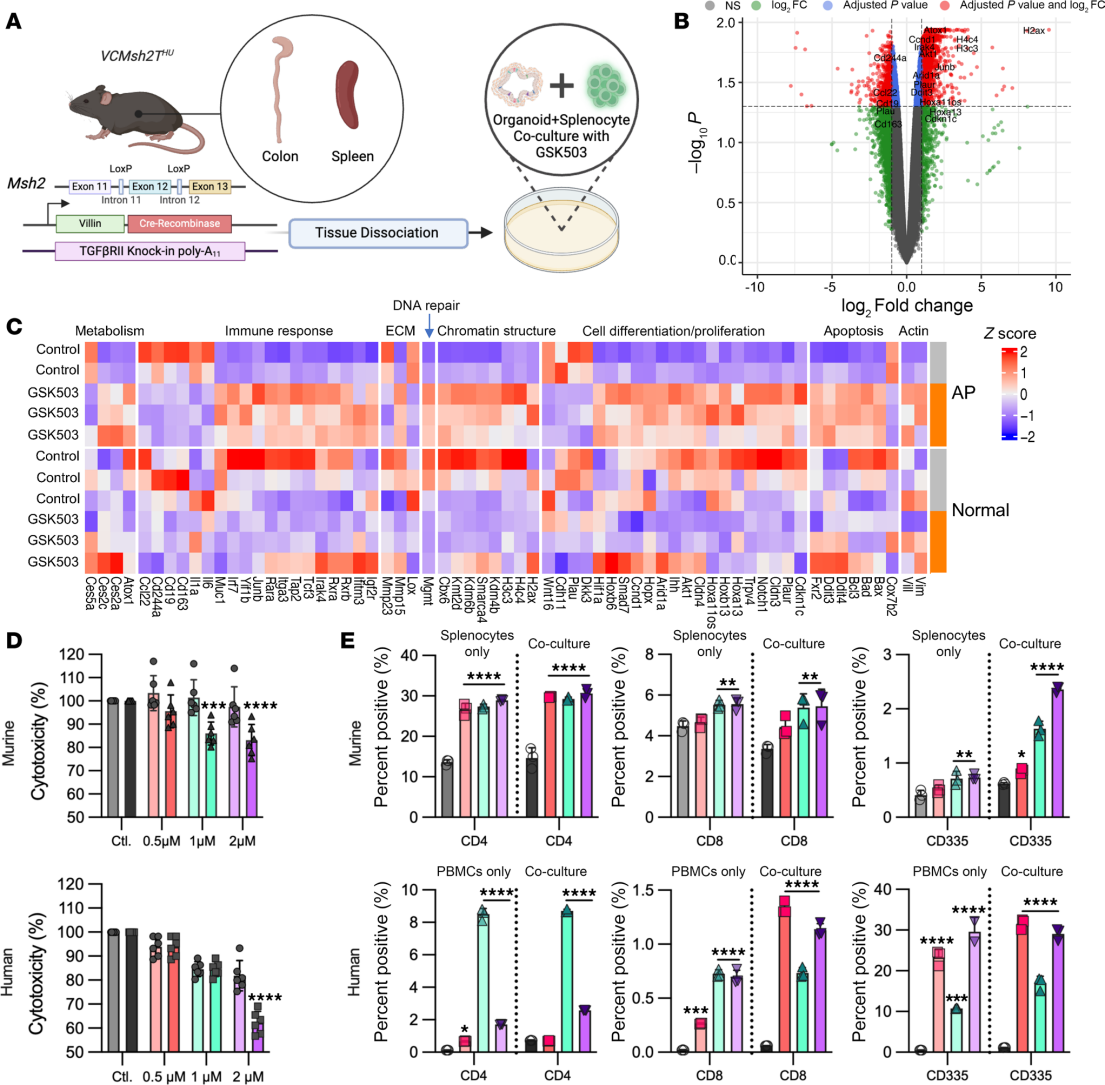
Inhibition of *Ezh2* enhances in vitro cytotoxic immune cell–mediated killing of murine and patient-derived organoids. (**A**) Schematic of the *VCMsh2T*^Hu^ murine model showing the workflow for coculture preparation. (**B**) Gene expression analysis of *VCMsh2T*^Hu^ organoids from GSK503-treated AP-MDOs is represented as a volcano plot, with log_2_FC on the *x* axis and −log_10_(adjusted *P*) on the *y* axis. Significant upregulated and downregulated genes (adjusted *P* ≤ 0.05, log_2_FC ≥ 3) are highlighted within pathways of interest. The horizontal line indicates Benjamini-Hochberg–adjusted *P* = 0.05. (**C**) A heatmap depicting gene expression changes in AP and NM organoids using logFC > 0.5 (FDR < 0.05). (**D**) Cytotoxicity results from murine (top) and human (bottom) organoid coculture experiments. Light-colored bars represent cytotoxicity in organoids treated with GSK503 (*n* = 6 organoid replicates), while dark-colored bars correspond to cytotoxicity in organoid coculture system treated with GSK503 (*n* = 6 coculture replicates). Gray bars represent controls, red bars represent 0.5 μM GSK503, cyan bars represent 1 μM GSK503, and purple bars represent 2 μM GSK503. Cytotoxicity data are normalized to controls and expressed as mean ± SEM from 3 technical replicates and 3 independent experiments. (**E**) Flow cytometric immune cell profiling in murine (*N* = 3, top) and human (*N* = 3, bottom) immune cells and cocultures following GSK503 treatment. Light-colored bars show immune cells from splenocytes (top) and PBMCs (bottom) alone, and dark-colored bars represent cocultures with or without GSK503. CD4 stains for helper T cells, CD8 for cytotoxic T cells, and CD335 for NK T cells. Statistical analyses were performed using 1-way ANOVA with multiple comparisons to the control (gray bars) and are presented as mean ± SEM from 3 biological replicates. *P < 0.05, ** *P* < 0.01, *** *P* < 0.001, **** *P* < 0.0001.

This study utilized a mouse model of LS, as described in the Methods and Figure 1A. To investigate the intrinsic transcriptomic changes induced by EZH2 inhibition with GSK503 (5 μM) in adenomatous polyps (AP) during tumorigenesis, we performed RNA-Seq analysis using mouse-derived organoids (MDOs) from large intestine AP (*N* = 2 controls, *N* = 3 treated) and adjacent normal colonic mucosa (NM; *N* = 3 controls, *N* = 3 treated) from *VCMsh2T*^Hu^ mice. The volcano plot in Figure 1B highlights differentially expressed genes (DEGs) in AP organoids following 5 μM GSK503 treatment, revealing 1,542 upregulated and 662 downregulated genes (log–fold change [logFC] > 0.5, FDR < 0.05) compared with untreated AP-MDOs. EZH2 inhibition selectively targeted precancers, with AP-MDOs showing a greater number of DEGs (Figure 1B, Supplemental Figure 1A, and Supplemental Table 1; supplemental material available online with this article; https://doi.org/10.1172/jci.insight.177545DS1), while NM exhibited a mosaic gene expression pattern (Figure 1C and Supplemental Figure 1B). In AP-MDOs, GSK503 altered expression patterns of genes involved in apoptosis, differentiation, chromatin structure, and immune response (Figure 1C). Furthermore, gene set enrichment analysis (GSEA) of DEGs in GSK503-treated AP-MDOs compared with nontreated AP-MDOs revealed several activated pathways, such as DNA replication, mitophagy, apoptosis, and p53 signaling (Supplemental Figure 2A). Conversely, the top suppressed pathways included cell adhesion molecules, ECM interactions, phototransduction, and complement and coagulation cascades (Supplemental Figure 2B).

To establish the optimal experimental dosing of GSK503, we tested the pharmacokinetics and dynamics of EZH2 inhibition using both *VCMsh2T*^Hu^ MDOs (Supplemental Figure 3, A and B) and single monolayer cultures of both microsatellite stable (MSS) and unstable (MSI) human cell lines (Supplemental Figure 3C). While GSK503 was the primary agent used throughout this study, tazemetostat, an alternative EZH2 inhibitor, was also compared head to head. Both EZH2 inhibitors reduced MDO viability by 50 percent at 15 μM (Supplemental Figure 3, A and B) and affected the viability of HCT116 (MSI-high) cells, but not SW620 (MSS) cells, at 10 μM (Supplemental Figure 3C). Dose-dependent changes in protein expression were assessed using Western blot analysis, showing that 1 μM GSK503 was sufficient to reduce H3K27me3 protein levels in MDOs (Supplemental Figure 3D).

We assessed organoid viability upon treatment with GSK503 in both *VCMsh2T*^Hu^ MDOs and patient-derived organoid (PDO) cocultures as an ex vivo proxy for immune-mediated cytotoxicity after a 24-hour treatment with 3 doses of GSK503 (0.5, 1, and 2 μM) (Figure 1D). Remarkably, both MDOs and PDOs showed a nonsignificant decrease in viability, even at 2 μM (*P* > 0.05, Figure 1D, light bars). However, MDOs cocultured with autologous splenocytes in the presence of 1 and 2 μM GSK503 exhibited statistically significant immune-mediated cytotoxicity compared with untreated controls (*P* < 0.0001 and *P* = 0.0005, respectively, Figure 1D, top). PDOs cocultured with patient-matched PBMCs showed significant cytotoxicity at 2 μM GSK503 (*P* < 0.0001, Figure 1D, bottom).

Flow cytometry was used to quantify the effects of GSK503 on T cell subpopulations within splenocytes and PBMCs. The addition of GSK503 (0.5, 1, and 2 μM) to murine splenocytes alone and MDOs cocultured with splenocytes significantly increased the abundance of CD4^+^ T helper cells (Figure 1E). CD8^+^ cytotoxic T cell levels were significantly increased compared with controls at both 1 μM (*P* = 0.002 for splenocytes only and *P* = 0.0087 for coculture) and 2 μM (*P* = 0.0024 for splenocytes only and *P* = 0.008 for coculture, Figure 1E and Supplemental Figure 4). While no significant differences were observed in the relative abundance of CD4^+^ and CD8^+^ T cells between the coculture system and splenocytes alone, these data suggest that EZH2 inhibition promotes direct immune cell proliferation. Additionally, we observed a dose-dependent increase in CD335^+^ NK cell abundance in the coculture system at 0.5 (*P* = 0.0176), 1 (*P* < 0.0001), and 2 μM (*P* < 0.0001) compared with nontreated controls (Figure 1E).

Complementary experimentation using PDOs cocultured with matched PBMCs recapitulated similar findings (Figure 1E). The addition of GSK503 to PBMCs alone and PDOs cocultured with matched PBMCs significantly increased the abundance of CD4^+^ T helper cells in both systems at 1 μM (*P* < 0.0001 for both PBMCs and coculture) and 2 μM (*P* < 0.0001 for both PBMCs and coculture) compared with nontreated controls (Figure 1E). Across all tested doses, GSK503 significantly increased the abundance of CD8^+^ cytotoxic T cells for both PBMCs alone and PDOs cocultured with PBMCs (*P =* 0.0001 for 0.5 μM PBMCs only; *P* < 0.0001 for all other data points, Figure 1E). Finally, we observed a significant increase in CD335^+^ NK cell abundance in both PBMCs alone and the coculture system at 0.5 μM (*P* < 0.0001 for both), 1 μM (*P* = 0.0004 for PBMCs only and *P* < 0.0001 for coculture), and 2 μM (*P* < 0.0001 for PBMCs only and coculture, respectively) compared with nontreated controls (Figure 1E).

### Inhibition of EZH2 reduces adenoma progression and promotes immune activation in VCMsh2T^Hu^ colorectal mucosa.

**Figure 2:**
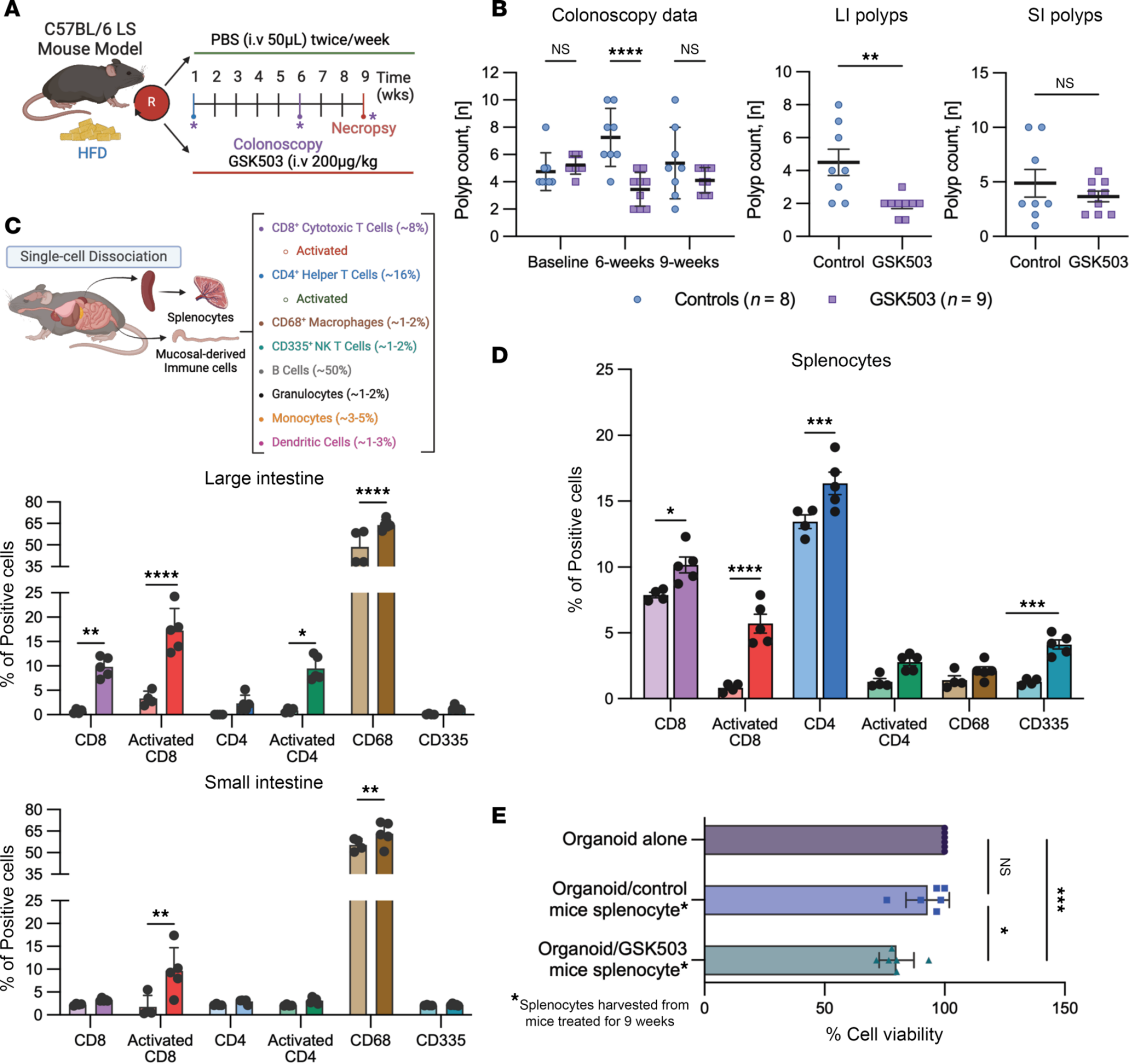
EZH2 inhibition reduces colonic polyposis and enriches adaptive immune cellularity in *VCMsh2T^Hu^* mice. (**A**) Flow chart of murine preclinical trial. (**B**) Polyp multiplicity in mice treated with GSK503 versus control. Colonoscopy data show no significant difference in polyp count at baseline (*P* = 0.4512) or after 9 weeks (*P* = 0.1558) but a significant reduction at the 6-week midpoint (*P* < 0.0001). Necropsy data show a significant decrease in colonic polyp multiplicity (*P* = 0.0045) in GSK503-treated mice, with no significant difference in small intestine polyps (*P* = 0.3706). LI, large intestine. Results are from 2 independent trials. (**C**) Flow cytometry results from the large intestine and small intestine of *VCMsh2T*^Hu^ mice from GSK503 preclinical trial (*n* = 4 control mice, *n* = 5 treated mice). Light-colored bars represent control mice, and dark-colored bars represent treated mice. Activated CD8^+^ T cells were significantly increased in both the large intestine (*P* < 0.0001) and small intestine (*P* = 0.0055) of GSK503-treated mice. GSK503 significantly increased macrophages (CD68) in the large intestine (*P* < 0.0001) and small intestine (*P* = 0.0062). Finally, GSK503 increased total CD8^+^ T cells (*P* = 0.0058) and activated CD4^+^ T cells (*P* = 0.012) in the large intestine only (*N* = 3 mice per condition). (**D**) Immune cell profiling from splenocytes harvested from mice in the preclinical trial (*n* = 5 mice). Light-colored bars represent control mice (*n* = 4), and dark-colored bars represent treated mice (*n* = 5). GSK503 significantly increased total CD8^+^ cells (*P* = 0.0103) and activated CD8^+^ T cells (*P* < 0.0001), total CD4^+^ T cells (*P* = 0.0007), and CD335^+^ NK cells (*P* < 0.001) compared with untreated controls (*N* = 3 mice per condition). (**E**) Autologous cocultures from 6 mice per treatment group. GSK503 treatment significantly reduced coculture MDO viability compared with that of untreated cocultures (*P* = 0.012) and organoids alone (*P* = 0.003). Statistical analyses were performed using Student’s *t* test (**B**–**D**) and 1-way ANOVA with multiple comparisons (**E**) and are presented as mean ± SEM. *P < 0.05, ** *P* < 0.01, *** *P* < 0.001, **** *P* < 0.0001.

A short-term 9-week preclinical trial was conducted using *VCMsh2T*^Hu^ mice treated with GSK503 (200 μg/kg body weight, via tail vein injection) (25) to assess in vivo effects of EZH2 inhibition on the mucosal immune microenvironment and its potential as an immune prevention strategy (Figure 2A). No signs of adverse events or significant differences in animal body weight were noted during the trial (Supplemental Figure 5). Murine colonoscopies performed at baseline indicated no significant difference in polyp multiplicity in the colon between treated and control mice (*P* = 0.451). After 6 weeks of GSK503 intervention, a significant reduction in colonic polyp multiplicity was observed in treated mice (*P* = 0.00003) compared with controls (Figure 2B). However, colonoscopies performed at 9 weeks, just prior to necropsy, did not show significant differences in polyp multiplicity between treated mice and controls (*P* = 0.1558, Figure 2B). Despite this, at necropsy, a significant reduction in polyp multiplicity was noted microscopically in treated mice compared with controls (*P* = 0.0045, Figure 2B). The discrepancy between colonoscopy and microscopic counts is likely due to differences in sensitivity between the two assessment methods and should be considered in data interpretation. Interestingly, no significant difference in polyp multiplicity in the small intestine (SI) was observed under the dissection microscope during necropsy (*P* = 0.3076, Figure 2B).

Changes to the immune landscape following GSK503 administration were assessed using flow cytometry on single-cell suspensions from spleen, large intestine, and SI tissues of *VCMsh2T*^Hu^ mice. This evaluation employed known immune cell markers, including CD134^+^ for CD4^+^ and CD137^+^ for subtypes of CD8^+^ T cells. GSK503 treatment resulted in a significant increase in both total and activated cytotoxic T lymphocytes (CD8^+^/CD137^+^, *P* = 0.0058/*P* < 0.0001, respectively), stromal macrophages (CD68^+^, *P* < 0.0001), and activated helper T lymphocytes (CD4^+^/CD134^+^, *P* = 0.0103) in the large intestine of *VCMsh2T*^Hu^ mice (Figure 2C). Additionally, GSK503 significantly increased activated cytotoxic T lymphocytes (CD8^+^, *P* = 0.0055) and stromal macrophages (CD68^+^, *P* = 0.0062) in the SI of *VCMsh2T*^Hu^ mice (Figure 2C). GSK503 also significantly increased the abundance of total and activated cytotoxic T lymphocytes (CD8^+^, *P* = 0.013 and < 0.0001, respectively), NK T lymphocytes (CD335^+^, *P* = 0.001), and total helper T lymphocytes (CD4^+^, *P* = 0.0007) in the spleen of *VCMsh2T*^Hu^ mice (Figure 2D). To assess sustained long-term immunity following GSK503 treatment, we established paired autologous MDOs cocultured with splenocytes from *VCMsh2T*^Hu^ mice after 9 weeks of GSK503 treatment. Consistent with above findings, MDOs cocultured with splenocytes from GSK503-treated mice exhibited a significant reduction in cell viability compared with MDOs alone (*P* = 0.003) and untreated coculture controls (*P* = 0.011, Figure 2E).

### Inhibition of EZH2 decreases markers of proliferation and stemness in VCMsh2T^Hu^ crypts.

**Figure 3:**
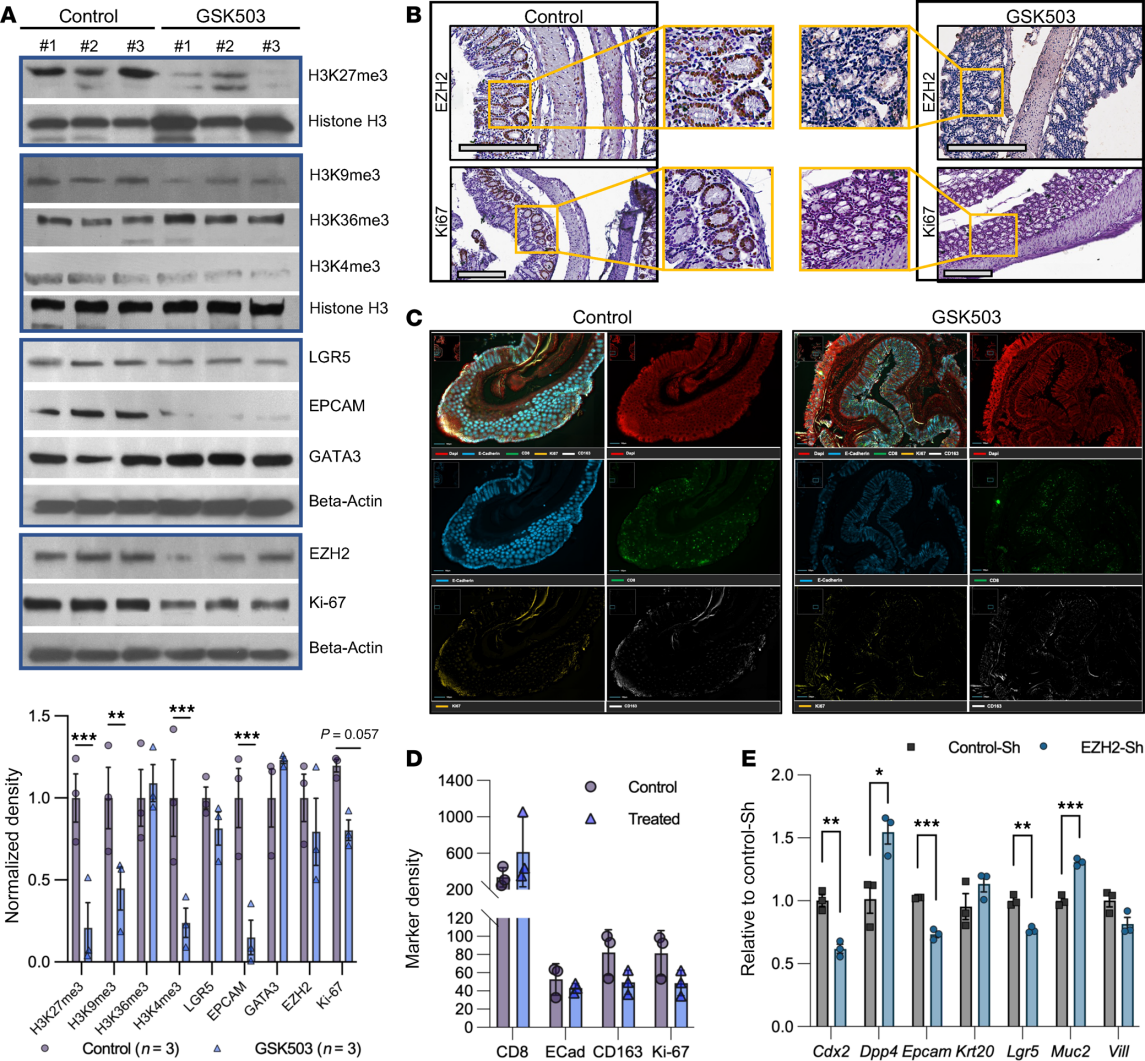
Inhibition of EZH2 promotes immune infiltration and activation in *VCMsh2T*^Hu^ colonic mucosa. (**A**) Western blot analysis was performed in the lysates from colonic mucosal stripping of *VCMsh2T*^Hu^ mice (*N* = 3/group): control and GSK treatment with quantification shown in bar graph below blots. Histone H3 modification was assessed using anti-H3K27me3 (*P* = 0.0003), anti-H3K9me3 (*P* = 0.0091), anti-H3K36me3 (*P* = 0.6534), and anti-H3K4me3 (*P* = 0.0005) antibodies with histone H3 as loading control. Stemness and proliferation were measured via anti-LGR5 (*P* = 0.3583), anti-EPCAM (*P* = 0.0001), anti-GATA3 (*P* = 0.2559), and Ki67 antibodies (*P* = 0.0572). Anti-EZH2 (*P* = 0.3091) was probed to assess efficacy of GSK503 activity in the colonic mucosa. The loading control for each nonhistone blot was β-actin. Quantification was performed using ImageJ, and density was normalized to control samples for each probe. (**B**) IHC staining of colonic tissue from *VCMsh2T*^Hu^ mice. Images shown are a single field of view (original magnification, ×20). Scale bar: 200 μm. (**C**) A representative image of sequential immunofluorescence using Lunaphore COMET platform from *VCMsh2T*^Hu^ colonic mucosa (*N* = 3/group) stained with DAPI (red), E-cadherin (blue), CD8 (green), Ki67 (yellow), and CD163 (white) (original magnification, ×20). (**D**) Quantification of Comet data shown in **C**. (**E**) EZH2 knockdown in mouse organoids phenocopied similar results obtained with GSK503 inhibition of EZH2. Quantitative gene expression analysis results demonstrated significant changes in gene expression for *Cdx*2 (*P* = 0.0026), *Dpp4* (*P* = 0.022), *Epcam* (*P* = 0.0003), *Lgr5* (*P* = 0.001), and *Muc2* (*P* = 0.0005). The mRNA levels of *Krt20* and *Vill* were not significant. The graphed data are expressed as mean ± SEM. For all graphs, Student’s *t* test was used to determine significance. *P < 0.05, **P < 0.01, ***P < 0.001, ****P < 0.0001.

Western blot analyses of protein lysates harvested from mucosal stripping of GSK503-treated *VCMsh2T*^Hu^ mice revealed intriguing alterations in histone marks. Specifically, there was a significant reduction in protein expression levels of H3K27me3 (*P* = 0.0003), H3K9me3 (*P* = 0.0091), and H3K4me3 (*P* = 0.0005), while no significant change was observed in H3K36me3 (*P* = 0.6534). The reduction of H3K27me3 levels indicated effective drug targeting by GSK503 in the colonic mucosa (Figure 3A). Furthermore, treated mice exhibited reduced mucosal proliferation (Ki67, *P* = 0.0572), a downtrend in stemness (LGR5, *P* = 0.3583), and a significant decrease in differentiation (EPCAM, *P* = 0.0001) compared with untreated mice (Figure 3A). These findings were further corroborated histologically through IHC analysis, which revealed reduced protein levels of EZH2 and Ki67 in the colonic mucosa of GSK503-treated mice (Figure 3B).

To overcome the limitations of conventional IHC in analyzing multiple markers simultaneously across different tissue sections, we employed high-plex immunofluorescence using the Lunaphore COMET platform. This method utilized a panel of 22 antibodies (Supplemental Table 2) on FFPE sections from control- and GSK503-treated mice (*N* = 3/group), targeting immune-oncology, lymphoid, myeloid, epithelial, and stromal markers, as described in Supplemental Methods. Treated mice exhibited a nonsignificant increase in CD8^+^ cells within the large intestine compared with control mice, suggesting that EZH2 inhibition promotes the recruitment or enrichment of colonic tissue–resident CD8^+^ T cells (Figure 3C). Quantitative analysis showed a decreasing trend in antiinflammatory cells (CD163^+^), proliferative cells (Ki67^+^), and epithelial cells (ECAD^+^, Figure 3D), though the results were not statistically significant. Levels of caspase-3 (an apoptotic marker), CD68 (a macrophage marker), and SPP1 (a cancer-associated fibroblast marker) showed nonstatistically significant decreasing trends, while other markers remained largely unchanged (Supplemental Figure 6, A and B).

To assess off-target and downstream effects of EZH2 inhibition, we generated an EZH2 knockdown MDO line using a lentivirus to express shRNA against *Ezh2* and scrambled shRNA (Control_sh) (Supplemental Figure 7, A and B). Levels of *Cdx2* (*P* = 0.0026), *Epcam* (*P* = 0.0003), and *Lgr5* (*P* = 0.001) mRNA expression decreased upon EZH2 knockdown, whereas the levels of *Dpp4* (*P* = 0.022) and *Muc2* (*P* = 0.0005) increased when compared with Control_sh MDO (Figure 3E). These results phenocopy our previous observations of decreased levels of LGR5 and EPCAM proteins shown by Western blot (Figure 3A).

### Inhibition of EZH2 increases gene expression of immune regulatory pathways in VCMsh2T^Hu^ crypts.

**Figure 4:**
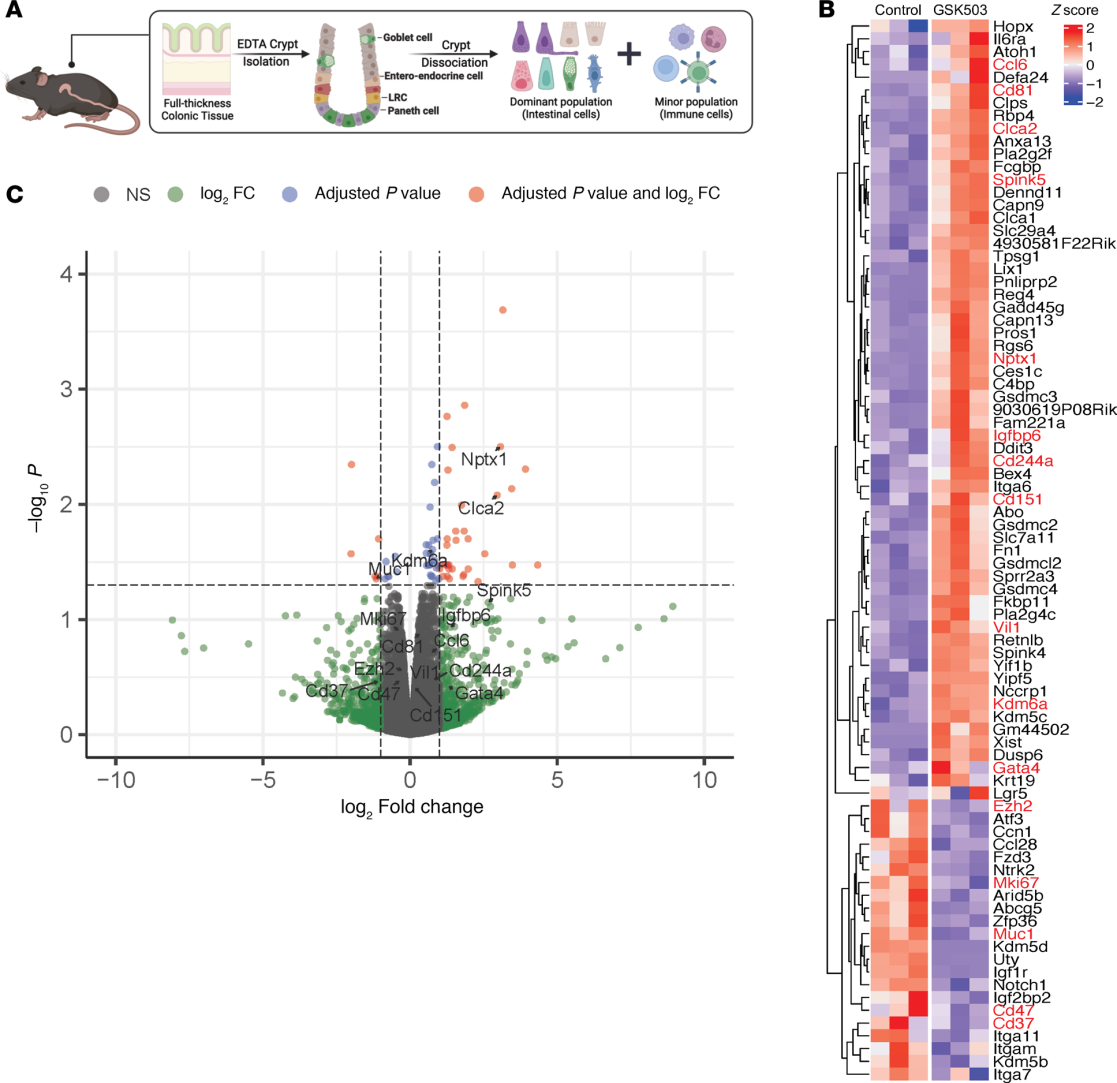
Inhibition of EZH2 increases expression of immune regulatory pathways in *VCMsh2T*^Hu^ crypts. (**A**) Schematic of single-cell preparation from *VCMsh2T*^Hu^ crypts for RNA-Seq. LRC, DNA-label-retaining cells. (**B**) Heatmap of differentially expressed [adjusted *P* < 0.05, absolute(logFC) > 1] genes in control- and GSK503-treated groups. Genes of interest are highlighted in red. (**C**) Genes from whole-transcriptome sequencing are displayed in volcano plots with log_2_FC on the *x* axis, and –log_10_(adjusted *P*) is shown on the *y* axis. The significant up- and downregulated genes with an adjusted *P* ≤ 0.05 and an absolute value of log_2_FC ≥ 1 highlight genes of interest, including tumor suppressor genes and NK-related genes. The horizontal line represents Benjamini-Hochberg–adjusted *P* = 0.05. The left and right vertical lines represent log_2_FC = ±1, respectively.

To examine the transcriptomic changes in colonic epithelium following EZH2 inhibition with GSK503, total RNA-Seq was performed using crypts isolated from GSK503-treated and control mice (Figure 4A). Our preparation of intestinal crypts predominantly contained intestinal cells, such as goblet, enteroendocrine, and Paneth cells, along with minor populations of immune cells. A heatmap and parallel Volcano plot of significant DEGs between control- and GSK503-treated mice are shown in Figure 4, B and C. Transcriptomic profiling identified 239 genes significantly dysregulated in the crypt fractions of treated mice, with 175 upregulated genes (log_2_FC > 0.25) and 64 downregulated genes (log_2_FC < –0.25, Figure 4C and Supplemental Table 3). A deeper analysis revealed upregulation of genes involved in tumor suppression (*Clca2*, *Spink5*, *Igfbp6*, *Nptx1*, *Bex4*, *Gadd45g*, *Yipf5*), immune regulation (*IL6ra*, *Ccl6*, *Cd81*, *Cd244a*, *Cd151*, and *Gata4*), apoptosis, and pyroptosis (*Ddit3*, *Rgs6*, and *Gsdmsc2/3/4*). Additionally, *Defa24*, a defensin expressed in Paneth cells, and *Reg4*, a protein expressed and secreted by crypts, were significantly upregulated in treated mice compared with controls. Notable downregulated genes included *Mki67*, *Ccn1*, *Muc1*, *Atf3*, *Ntrk2*, *Arid5b*, *Igf1r*, *Igf2bp2*, *Cd47*, and *Cd37* (oncogenic function) and integrin-related genes, *Itga7*, *Itga11*, *Itgam* and *Notch1* (cancer stem cell markers). Interestingly, epigenetic regulators *Kdm6a* (lysine 27 demethylase) and *Kdm5c* (lysine 4 demethylase) were upregulated, while *Ezh2* (lysine 27 methyl transferase) was downregulated. These findings align with our Western blot and IHC analyses, demonstrating decreased levels of histone modification at H3K27 and EZH2 proteins in crypts of treated mice (Figure 3A).

Using in silico XCELL analysis, we explored immune cell abundance following EZH2 inhibition through transcriptomic changes in immune cell markers from RNA-Seq data (Supplemental Figure 8A). This cell deconvolution analysis showed a significant increase in granulocyte-monocyte progenitor cells (*P* = 0.0009), immature T cells (*P* = 0.036), and stroma-related cells, as measured by stroma score (*P* = 0.0417). These results support the flow cytometry analysis presented in Figure 2B. GSEA using DEGs for WikiPathway genes highlighted the activation of cholesterol biosynthesis, aerobic glycolysis, and deregulation of RAB and RAB effector genes in bladder cancer (Supplemental Figure 8B, top). Suppressed pathways included the FXR pathway (inflammation, metabolism, and cholesterol synthesis), the PXR pathway (cancer stem cells in colon cancer and chemoresistance), and the PRC2 interactome (Supplemental Figure 8B, bottom).

**Figure 5:**
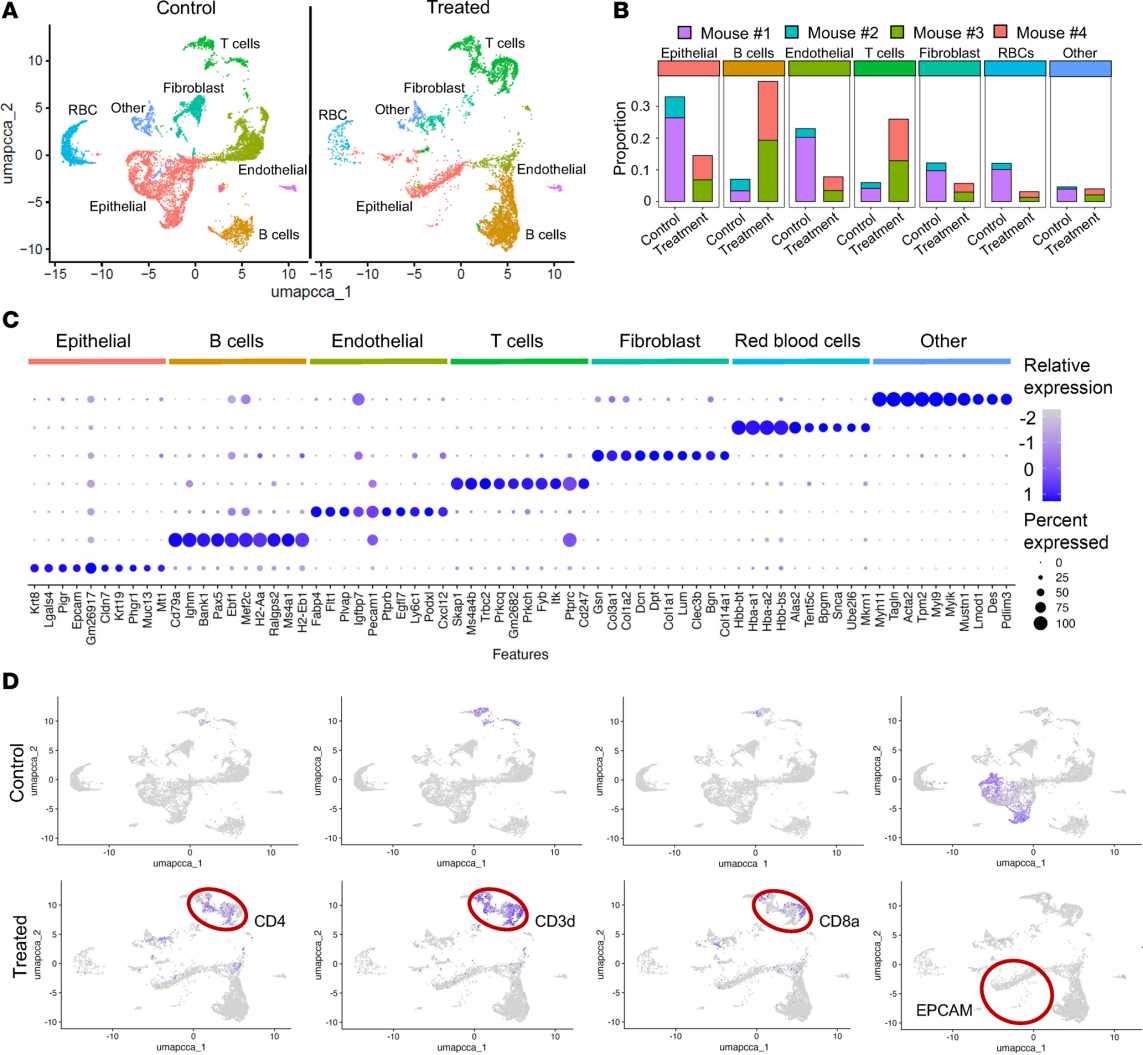
Inhibition of EZH2 increases immune cellularity in *VCMsh2T*^Hu^ crypts. (**A**) UMAP results from single-cell RNA-Seq analysis of single-cell suspension of colon harvested from GSK503-treated and control mice (*N* = 2 mice/group). (**B**) Bar graph depicting UMAP results as proportion of cells (*y* axis) in control versus treated mice. (**C**) Dot plot stratified by cluster as noted by labeled colored bar above each plot. Circle size correlates to percentage of gene expression. Circle color denotes relative gene expression. (**D**) UMAP results showing enrichment of immune cell populations and a decreased abundance of epithelial cells in GSK503-treated mice, as highlighted by the red circles.

Single-cell RNA-Seq from control (*n* = 2) and treated (*n* = 2) mice helped elucidate dynamic changes in the cellular landscape within murine crypts following GSK503 treatment. We profiled a total of 42,631 cells from the colon with the UMAP plot demonstrating distinct separation of cells based on known markers (Figure 5A); a heatmap of genes in each cluster is shown in Supplemental Figure 8C. K-means clustering, an unsupervised algorithm, grouped cells into 6 major clusters and 1 other cluster, yielding a total of 7 unique clusters. Notable markers included *Krt8*, *Epcam*, and *Cldn7* in the epithelial cell cluster; *Cd79a* and *Ighm* in the B cell cluster; *Fabp4* and *Flt1* in the endothelial cell cluster; *Skap1* and *Trbc2* in the T cell cluster; *Gsn* and *Col3a1* in the fibroblast cluster; *Hbb-bt* and *Hba-a1/a2* in the red blood cell (RBC) cluster; and *Myh11* and *Tagln* in the “Other” cell cluster (Figure 5, A and B). A dot plot depicts predominantly expressed genes stratified by cell cluster in GSK503-treated mice compared with control mice (Figure 5C). These data also show a notable shift in cell populations, with an increase in infiltrating immune cells, including CD3d^+^, CD8a^+^, and CD4^+^ T cells, and a decrease in EPCAM^+^ cells in the colon of GSK503-treated mice (Figure 5D). These data also show a notable shift in cell populations, with an increase in infiltrating immune cells, including CD3d^+^, CD8a^+^, and CD4^+^ T cells, and a decrease in EPCAM^+^ cells in the colon of GSK503-treated mice (Figure 5D), further supporting our Western blot analysis (Figure 3A) and Ezh2 knock-down data (Figure 3E).

### EZH2 inhibition decreased H3K27 methylation and increased promoter activation in LS mouse genome.

**Figure 6:**
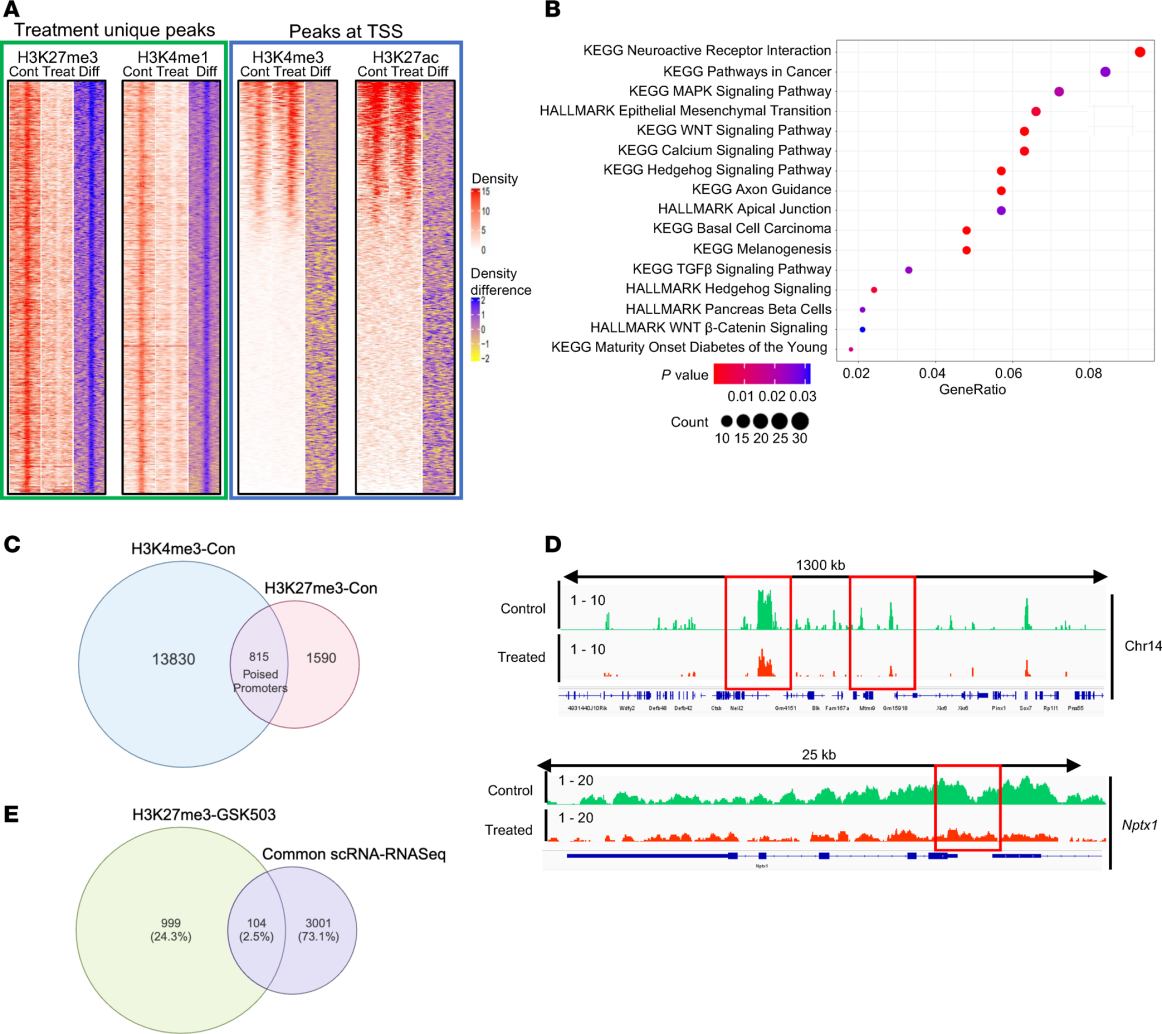
EZH2 inhibition decreases the occupancy of H3K27me3 and H3K4me1 in *VCMsh2T^Hu^* mice treated with GSK503. (**A**) ChIP-Seq density heatmaps in control (cont) and GSK-treated (treat) groups and their difference (diff), ranked by methylation read intensity within ±1.0 kb (H3K27me3 and H3K4me1, green box) of peak summits and ±1.0 kb from TSS for H3K4me3 and H3K27ac (blue box). (**B**) GSEA of H3K27me3-enriched genes in control mice showing pathway activation. (**C**) Venn diagram showing the occupancy of H3K4me3 and H3K27me3 in genes of the poised promoters in control mice. (**D**) Representative histone methylation level at chromosome 14 and the *Nptx1* gene. (**E**) Venn diagram of shared upregulated genes in both scRNA-Seq and RNA-Seq data sets compared with H3K27me3 occupancy in GSK503-treated mice.

Given that GSK503 treatment decreased levels of EZH2 and its activity on K27 methylation, we aimed to determine the abundance of H3K27me3 within specific chromatin fragments in colonic crypts from GSK503-treated *VCMsh2T*^Hu^ mice compared with controls. Additionally, we assessed the presence of bivalent occupancy of H3K4me3 (a mark of promoter activation) and H3K27me3 in control *VCMsh2T*^Hu^ mice as a proxy for poised promoter activity. Heatmaps from the ChIP-Seq analysis clearly showed a global reduction in H3K27me3 peak height within ±1 kb of chromatin fragments in GSK503-treated mice compared with controls (Figure 6A). Our analysis identified 2,840 peaks corresponding to H3K27me3 in control mice and 1,079 peaks in treated mice, thus indicating a marked reduction of K27 methylation upon treatment with GSK503 (Table 1 and Supplemental Tables 4 and 5). Notably, we did not observe an increase in H3K4me3 peaks — histone marker for promoter activation — in treated mice compared with controls (14,219 vs. 14,175 peaks; Table 1, Figure 6A, and Supplemental Tables 6 and 7). GSEA of H3K27me3-enriched genes in control mice revealed associations with several key pathways, including cancer, EMT, WNT, calcium, and hedgehog signaling (Figure 6B). Our data indicated that 815 genes contained a bivalent occupancy of both H3K4me3 and H3K27me3 within the promoter regions in control mice (Figure 6C), suggesting that the presence of H3K27me3 inhibits transcriptional activation.

### EZH2 inhibition reprograms histone methylation enhancer marks, H3K4me1 and H3K27ac, in the genome of LS mice.

Active enhancer or superenhancer elements are *cis*-regulatory DNA elements characterized by the presence of H3K4me1 and H3K27Ac; they play a pivotal role in driving oncogene expression during tumor progression and development. However, the landscape of these enhancer marks in MMRd LS carcinogenesis remains largely unexplored. We utilized the *VCMsh2T*^Hu^ mouse model to delineate the enhancer landscape and assess the effects of EZH2 inhibition on reprogramming enhancer marks of H3K4me1 and H3K27Ac within murine crypts.

Consistent with our findings for H3K27me3, the number of genes associated with H3K4me1 was substantially greater in control mice (11,319 genes) compared with treated mice (5,606 genes), with 16,282 H3K4me1 peaks detected in control mice versus 7,464 in treated mice (Table 1, Figure 6A, and Supplemental Tables 8 and 9). This indicates a nearly 50 percent reduction in H3K4me1 marks in GSK503-treated mice compared with controls. In contrast, the number of H3K27ac marks, a marker for super enhancer enrichment, was higher in the treated group (11,312 vs 10,515 peaks) compared with controls, with 8,805 genes in treated and 8,346 genes in control group (Table 1, Figure 6A, and Supplemental Tables 10 and 11).

Figure 6D presents two snapshots of chromatin fragments within the genomic loci of chromosome 14 and a separate tracing of the *Nptx* gene using Integrated Gene Viewer (IGV). Both tracings revealed decreased H3K27me peak amplitude in treated mice (red peaks) compared with controls (green peaks). Notably, the *Nptx* gene, a tumor suppressor, was upregulated in treated mice, as indicated by RNA-Seq analysis (Figure 4C), suggesting that its reactivation is mediated by GSK503 treatment through removal of H3K27 methylation. Additionally, the IGV snapshot of H3K4me1, H3K27Ac, and H3K4me3 levels within a 1.6 Mb genomic fragment of chromosome 6 illustrates the differential levels of these histone modifications between control and treated mice (Supplemental Figure 9). To explore the relationship between upregulated genes and H3K27me3 histone modifications upon GSK503 treatment, we compared single-cell RNA-Seq (scRNA-Seq) and RNA-Seq data with ChIP-Seq results. The data showed that 9,964 genes were upregulated in the scRNA-Seq analysis and 5,802 genes in the RNA-Seq analysis, with 3,105 upregulated genes shared across both platforms (Supplemental Figure 10A). Of the 9,964 upregulated genes in scRNA-Seq, 290 were occupied by low levels of H3K27me3 due to GSK503 treatment, while 225 of the 5,802 upregulated genes in RNA-Seq showed H3K27me3 occupancy (Supplemental Figure 10, B and C). Of the 3,105 upregulated genes shared between scRNA-Seq and RNA-Seq, 104 genes exhibited low H3K27me3 occupancy due to GSK503 treatment in both datasets, thus suggesting that EZH2 inhibition may lead to their upregulation (Figure 6E).

### Human LS tumors express high levels of EZH2.

To evaluate the translational relevance of targeted EZH2 methyltransferase inhibition in LS, we first analyzed *EZH2* mRNA expression in normal mucosa, adenoma (precancer), and adenocarcinoma samples from patients with LS treated at the University of Texas MD Anderson Cancer Center (MDACC) using RNA-Seq data. The results showed a stepwise increase in *EZH2* mRNA expression with advancing pathology, showing a significant elevation in adenomatous and neoplastic tissue compared with adjacent normal tissue (*P* = 0.024 and 0.001, respectively; Supplemental Figure 11A). IHC staining results further supported this trend, revealing low EZH2 protein expression in normal mucosa, moderate levels in adenomas, and higher expression in adenocarcinoma tissue, although these differences were not statistically significant (Supplemental Figure 11B). These findings, combined with existing literature, underscore the potential of EZH2 inhibition as a chemopreventive strategy in LS-associated CRC, warranting further investigation.

## Discussion

To our knowledge, no previous study has explored the use of an EZH2 inhibitor as a potential immune-modulating agent for LS carcinogenesis. However, immune interception strategies have been widely studied, focusing on developing immune-priming agents that can serve as adjuvants prior to vaccination to target specific neoantigens (10). A recent clinical trial from our group demonstrated that daily low doses of naproxen (both 220 and 440 mg) improve cytotoxic T cell enrichment in the normal mucosa of patients with LS (23, 28). Building on this discovery, a preclinical trial using the *VCMsh2* mouse model showed that immunization with a frameshift peptide (FSP) vaccine elicited robust antitumor activity and improved long-term survival in mice receiving daily naproxen along with neoantigen vaccination (29). While naproxen may enhance immune enrichment, our study provides rationale for using EZH2 inhibitors as a more effective adjuvant that not only increases immune cellularity but also initiates immune cell activation to elicit cytotoxic effects.

In fact, a study by Zingg et al. demonstrated that EZH2 overexpression silences immunogenicity and antigen presentation in cancers such as melanoma, which serves as a gold standard for studying neoantigen biology in tumors (30). Functional studies indicated that inhibiting EZH2 with GSK503 reversed several resistance mechanisms in melanomas, leading to improved synergy between different immunotherapies and enhanced antitumor cytotoxicity (30). Our study observed that inhibiting EZH2 enriched T cell activity and increased the abundance of effector CD8^+^ T cells intratumorally. While this has been observed in melanoma, other studies found that CRC cells exhibit similar repression of CD8^+^ cytotoxic T cell trafficking to tumor sites, suggesting that the epigenetic silencing of immune pathways is a vital mechanism in CRC biology (10, 31).

Consistent with prior data using subtoxic doses of GSK503 as an immune modulator, our study indicates that targeting EZH2 in a murine model of LS promotes robust T cell–mediated cytotoxicity both in vitro and in vivo. In cocultures of normal murine mucosa organoids and autologous splenocytes, GSK503 stimulation induced marked cytotoxicity, which may partially be explained by immune recognition of neoantigen expression in MMRd cells within the normal mucosa, as previously shown in studies using the VCMsh2 model (29). To further explore this, we utilized flow cytometry to quantitatively assess changes in immune cell abundance — both locally and systemically — following GSK503 treatment, revealing a substantial increase in immune cell abundance and activation in the in vitro coculture system.

In a preclinical trial using the *VCMsh2T*^Hu^ murine model, we corroborated our in vitro results in vivo. The data revealed a striking reduction in viability in the autologous coculture system from mice treated with GSK503. Additionally, these mice showed robust enrichment of immune cells within the mucosal tissues. Specifically, GSK503 treatment increased distinct populations of cytotoxic T cells and B cells, likely enhancing immune surveillance and activity. This shift in immune cell composition underscores the potential of EZH2 inhibitors to reshape the immune landscape toward an antitumor phenotype.

The observed reduction in epithelial and endothelial cells — often involved in forming the structural components of tumors — may create a less favorable environment for tumor growth, contributing to decreased polyp multiplicity observed with GSK503 treatment. Similarly, the decrease in fibroblast cells, known to support tumor progression and metastasis, further indicates a disruption of the tumor-supportive microenvironment. By disrupting the immunosuppressive environment maintained by EZH2, its inhibition can promote a more effective antitumor immune response.

Our study found that inhibiting EZH2 reduces proliferation and differentiation in *VCMsh2T*^Hu^ crypts, suggesting that the loss of differentiated cells in treated mice results from activated apoptosis and reduced survival pathways, along with immune activation of regulatory T cells in the crypts compared with controls. We also observed an enriched immune cell population in the crypt fractions of treated mice. GSK503 treatment downregulated EZH2 at both the protein and mRNA levels while upregulating *Kdm6a* (lysine 27 demethylase), indicating that the global reduction of H3K27me3 is due to combined EZH2 inhibition and *Kdm6a* upregulation. Additionally, the reduction in H3K4me1 levels in treated mice may be linked to decreased expression of *Kmt2c* and *Kmt2d*, methyltransferases responsible for H3K4me1 enhancer marks. These findings provide mechanistic insight into the epigenetic regulation of H3K27me in LS carcinogenesis and further our understanding of EZH2 inhibition as a potential chemopreventive strategy.

We acknowledge several limitations of this study that warrant further investigation. First, our MDO and PDO coculture systems did not include organoids derived from apparent polyp tissue. However, the observed cytotoxic effects may have been mediated by T cell recognition of neoantigens presented by MHC-I molecules before polyp formation. This is supported by recent data showing that *VCMsh2* mice develop aberrant FSPs, which can be targeted with FSP-based vaccines to enhance T cell immunity (29). While intriguing, evaluating this interaction is beyond the scope of this study but remains an important area for future research.

Additionally, our inability to assess the SI during the preclinical trial prevented in vivo measurement of SI polyp multiplicity. The lack of significant differences between groups may stem from the abundance of Peyer’s patches, which could influence immune cellularity. Furthermore, SI polyps are a common off-target effect in CRC models and may be unrelated to our findings, though further characterization of this mouse model is warranted.

Another limitation is the small sample size of human LS colorectal tissues, which restricts the translational relevance of our findings. However, RNA-Seq of LS colorectal samples revealed a significant, stepwise increase in EZH2 expression across advancing tissue histology, highlighting its potential role in LS tumorigenesis. Further studies are needed to clarify EZH2’s function and therapeutic relevance in this context.

Finally, we cannot definitively determine whether the antitumor effects of EZH2 inhibition result from direct effects on neoplastic epithelial cells or indirect effects through immune cell reprogramming. While our data indicate significant changes in immune cellularity, immune-related gene expression, and epigenetic modulation following EZH2 suppression, the role of tumor-immune interactions remains unresolved. Additionally, we observed key differences between pharmacologic inhibition and genetic suppression of EZH2 — while both approaches reduced LGR5 and EPCAM levels, only genetic suppression upregulated select differentiation markers, suggesting broader transcriptional changes. To systematically assess these differences and distinguish direct antitumor effects from indirect consequences, RNA-Seq followed by scatter plot analysis could provide deeper insight into EZH2’s role in tumor cell differentiation and therapy response. Further studies, including immune depletion experiments, will be essential to dissect these mechanisms and refine EZH2-targeted therapeutic strategies.

In conclusion, effective EZH2 inhibitors have been tested in several clinical trials for the treatment of both solid and liquid tumors with the aim of abrogating tumorigenicity, but none have explored this agent class in LS (32). Thus, this work establishes a preliminary framework for developing a phase I clinical trial using an EZH2 inhibitor as an immunoprevention strategy for patients with LS, either alone or in conjunction with immunization using a neoantigen vaccine, which is currently being pursued in various animal models in the laboratory.

## Methods

### Sex as a biological variable.

For all murine studies, both female and male mice were included, ensuring a 50:50 distribution. Clinical samples were selected to ensure equal representation of male and female patients from the MDACC biorepository.

### Mice.

C57BL/6J (RRID:IMSR_JAX:016231) conditional knockout mice for the MMR gene *Msh2* (*Msh2^LoxP/LoxP^*) were crossed with *Villin-Cre*–transgene mice (RRID:IMSR_JAX:004586), generating *VCMsh2^LoxP/LoxP^* mice (33). These were further crossed with an in-house knockin humanized *Tgf*β*RII* model referred to as *VCMsh2^LoxP/LoxP^*. This humanized knockin introduces an 11 poly (A) microsatellite repeat in the *Tgf*β*RII* gene, leading to the development of 1–3 colorectal adenomas and stage I CRCs after 8 months of age when fed with a high-fat diet, similar to other models developed in-house (34). To expedite polyp formation, from 8 weeks of age, mice were continuously fed a Western-style diet known to increase reactive oxygen species production and DNA damage (35). This diet consisted of 20% fat, 0.5 calcium mg/g, 0.11 vitamin D_3_ IU/g, 3.6 PO_4_ mg/g, 2% fiber, 0.23 folic acid μg/g, 0.3% I cysteine, 0.12% choline bitartrate, and approximately 4.5 kcal/g (Newark Stress Diet C, D16378Ci, Research Diets Inc.).

Mice were randomized into two groups: GSK503 (EZH2 inhibitor) and control (vehicle). Tail vein injections were administered biweekly using a restraining apparatus and sterile technique, with GSK503 (Selleckchem) given at 200 μg/kg body weight (50 μL injection volume) in sterile PBS, while control mice received sterile 20% captisol in PBS. Body weight was monitored bimonthly, and animal behavior was observed daily.

### Murine tissue preparation.

*VCMsh2T*^Hu^ mice from both cohorts — treatment and control — were humanely sacrificed at the completion of the preclinical drug intervention trial. Tissues were collected and snap frozen in liquid nitrogen or placed in formalin for paraffin embedding.

### Murine colonoscopy.

*VCMsh2T*^Hu^ mice were anesthetized with isoflurane in accordance with standard MDACC IACUC practices. Positioned prone on a heating pad, 1 mL sterile PBS was administered via a stainless gavage cannula to flush feces from the colon. A 1 mm rigid bore proctoscope (Karl Storz) was gently advanced through the colon under video guidance. The scope was slowly retracted, providing visual coverage of the colon from the cecum to the anal verge. Polyps were manually counted during the endoscopy procedure. Endoscopy was well tolerated, and no adverse events were noted.

### Tissue IHC analysis.

Murine tissue sections were cut from FFPE Swiss rolls at 5 μm and deparaffinized using a series of ethanol washes beginning with Histo-clear (National Diagnostics) followed by 100%, 95%, 70%, and 50% ethanol. Antigen retrieval was performed in sodium citrate buffer (pH 6) using a hot plate. Slides were blocked for 1 hour using 20% goat serum in PBS. Slides were incubated overnight with the following primary antibodies in 5% goat serum in PBS: anti-EZH2 (Cell Signaling Technologies, 5246S, dilution 1:500) and Ki67 (Biolegend 652402, dilution 1:1,000). Slides were then incubated in secondary antibody (ImmPRESS secondary antibody [HRP polymer] was used depending on the primary source; goat anti-rabbit IgG was used as EZH2 primary antibody [Vector Laboratories, MP-7451]; goat anti-mouse IgG was used as Ki-67 secondary antibody [Vector Laboratories, MP-7452]) for 1 hour at room temperature and then exposed to DAB (Vector Laboratories) for 1 minute followed by Mayer’s hematoxylin counterstain for 1 minute. Slide were imaged using an Aperio scanner (Leica Biosystems). All clinical samples were processed and prepared by the pathology core at MDACC. IHC quantification was performed using the H-score method, defined as the ratio of the weighted sum of the number of positive cells to the total number of detected cells. Slides were analyzed and scored by an expert pathologist at MDACC.

### Crypt organoid preparation and culture.

Freshly isolated colons from *VCMsh2T*^Hu^ mice were used for organoid preparation. The colon was flushed with PBS to wash feces and undigested food and incised longitudinally. Tissue samples were cut into 2–5 cm pieces, washed with PBS 3 times to remove remaining contaminants, such as hair and feces, followed by incubation in 2 mM PBS/EDTA on ice for 5 minutes. Tissue pieces were subsequently incubated in 2 mM PBS/EDTA on a rotating platform at 4°C for 1 hour. Vigorous shaking yielded free crypts, and this process was repeated with 5 mM PBS/EDTA for 30 minutes. Isolated crypts were pelleted at 600*g* for 5 minutes and resuspended in Matrigel (Corning, catalog 356234) and dispensed as 50 μL domes into 4 cm petri dishes. After Matrigel polymerization, the embedded cells were overlaid with 4 mL CMGF++ media per petri dish, as described: Advanced DMEM/F12 supplemented with penicillin/streptomycin (Thermo Fisher Scientific, catalog 15140122), 10 mM HEPES, 2 mM GlutaMAX, 1 × B27 (Invitrogen), 1 × N2 Supplement (Invitrogen, catalog 17502048), 100 μg/mL Primocin (Invivogen, catalog ant-pm-05), 10 mM Prostaglandin E2 (Selleckchem), 10 mM Nicotinamide (MilliporeSigma), 10 nM gastrin I (MilliporeSigma), and 1 mM N-acetylcysteine (Wako), 50 ng/mL mouse recombinant EGF (Life Technologies), 100 ng/mL mouse recombinant Noggin (Peprotech, catalog 120-10C), 0.1% 250 μg/mL Amphotericin B (Gibco, catalog 15290018), Noggin/R-Spondin/Wnt-3A conditioned medium (in-house cell line), 500 nM A83-01 (Tocris, catalog 2939), and 10 μM SB202190 (MilliporeSigma, catalog S7067). A 10 μM Rock inhibitor, Y-27632 (MilliporeSigma, catalog no. Y0503), was added to the culture medium upon initial passage and freezing. Organoids were cultured with fresh media every 2–3 days in an incubator at 37°C in 5% CO_2_ and passaged every 7–10 days.

### Isolation of intraepithelial and lamina propria lymphocytes.

Aseptically removed colon and SIs were flushed with PBS to wash feces and undigested food and incised longitudinally. Tissue samples were cut into 2–3 cm pieces and washed with PBS 3 times to remove remaining contaminants. The prepared tissue samples were then immersed in a dissociation media consisting of 1 mg/mL collagenase IV, along with Advanced DMEM/F12, HEPES (Thermo Fisher Scientific) supplemented with penicillin/streptomycin (DFH media). The dissociation process was aided by homogenization using a glass dounce homogenizer tube and applying a grid plunging technique. The resulting content was transferred to a 50 mL canonical tube, combined with 5 mL dissociation media and 15 mL DFH media mixture and subsequently incubated in a benchmark mixer at 37°C for 1 hour. Following incubation, the tissue pieces were filtered through 70 and 40 μm filters to isolate single cells while removing tissue debris and aggregates. The filtered samples were then washed twice with ice-cold PBS and, subsequently, centrifuged at 600*g* for 5 minutes at 4°C to pellet the isolated immune cells. The pelleted cells were resuspended in an appropriate cell staining media to preserve their viability and antigenic characteristics for downstream immune cell analysis using flow cytometry.

### Isolation of splenocytes.

Aseptically harvested spleens from *VCMsh2T*^Hu^ mice were collected in 15 mL tubes containing 5 mL ice-cold RPMI/10% FBS or balanced salt solutions (BSS)/10% FBS. To generate a single-cell suspension, the spleen was placed between two pieces of a sterile 100 μm cell strainer mesh in a petri dish containing 2 mL ice-cold RPMI/10% FBS or BSS/10% FBS. The plunger of a 1 mL syringe was used to mash the organ until homogenously torn into small fragments. The cell suspension was transferred to a 15 mL tube, and the mesh was washed with ice-cold RPMI/10% FBS or BSS/10% FBS. The remaining cell suspension was collected, added to the same 15 mL tube, and centrifuged at 453*g* for 5 minutes at 4°C. After removing the supernatant, the pellet was resuspended in 5 mL RBC lysis solution (Qiagen, catalog 158106) and incubated for 5 minutes at room temperature. RBC Lysis reactions were stopped by adding 14 mL ice-cold BSS/10% FBS and centrifuged at 453*g* for 5 minutes at 4°C. Splenocytes were cultured with R10 media consisting of RPMI supplemented with 10% FBS, 1% L-glutamine, 1% penicillin/streptomycin, 1% 1 M HEPES buffer, and 330 U/ml IL-7 (R&D Systems, catalog no. 207-IL-005) for 3 days. On day 3, cells were transferred to the coculture experiment.

### Mouse organoid and splenocytes coculture experiment.

Harvested *VCMsh2T*^Hu^ organoids were suspended in Matrigel and dispensed as 50 μL domes into a 24-well plate. After Matrigel polymerization, organoids containing Matrigel dome were overlaid with 500 μL CMGF++/R10 media mixture containing autologous splenocytes (1:1 ratio). The EZH2 inhibitor GSK503 was added to the treatment group containing organoids, splenocytes, and organoid and splenocytes coculture at the concentration of 0.5, 1, and 2 μM (in DMSO). Untreated wells served as controls. After 72 hours of incubation, organoids were collected by gently scrapping the dome. Collected organoids dome were dissolved using cell recovery solution (Corning, catalog CLS354270), incubated on ice for 1 hour, and then pelleted at 390*g* for 5 minutes at 4°C for downstream viability analyses. The splenocytes were collected by gently scraping the surface of the plate and then aspirating the supernatant for flow cytometry analyses.

### Organoid viability assay.

After 72 hours of incubation with GSK503, the cell viability was assessed using CellTiter-Glo 3D Cell Viability Assay (Promega, catalog G9682). Organoids collected from cocultures and alone assays were placed separately in single wells of a 96-well opaque culture plate (BD Falcon, catalog 3603). CellTiter-Glo 3D reagent was added to each well, and the luminescence signal was read after 30 minutes with the bioluminescence reader following the manufacturer’s guidelines. Similarly, the effects of GSK503 and tazemetostat on cell viability were tested in HCT116 (ATCC CCL-247; microsatellite instable [MSI]) and SW620 (ATCC CCL-227; MSS) cell lines after 72 hours of treatment with serial doses of each EZH2 inhibitor using CellTiter-Glo for 2D cultures.

### Western blot.

The mucosal lining of *VCMsh2T*^Hu^ mice treated with EZH2 inhibitor GSK503 (*n* = 3 per group) and controls (*n* = 3 per group) was stripped for subsequent analysis. The tissue was homogenized in radioimmune precipitation assay buffer with complete Mini protease and PhosSTOP phosphatase inhibitor (Roche Diagnostics, catalog 4906845001). Lysates were centrifuged, supernatants were collected, and the total protein concentration in lysates was assessed using the BCA protein assay kit (Pierce Biotechnology, catalog 23225). The lysates were separated on polyacrylamide gels (Bio-Rad) for Western blot analysis. The following primary antibodies were used: anti-H3K27me3 (Diagenode, 069-050, 1:2,000), anti-H3K36me3 (Diagenode, 058-050, 1:2000), anti-H3K9me3 (Upstate Cell Signaling Solutions, 07-422; 1:500), anti-H3K4me3 (Active Motif, 39159; 1:1,000), anti-EZH2 (5246S, 1:1,000), anti-EPCAM (Cell Signaling, 3599S, 1:1,000), anti-H3 (ab8898-100, 1:1,000), anti-GATA3 (ab215216; 1:1,000), anti-LGR5 (TA503316, OriGene; 1:1,000), anti-Ki67 (Biolegend, 652402, 1:1,000), and anti–β-actin (Cell Signaling; 1:5000). Goat anti-mouse (1:6,000) or goat anti-rabbit (1:6,000) secondary antibodies were used (Abcam). The membranes were detected using the Clarity Western ECL Substrate Kit (Bio-Rad, catalog 1705061). Quantification was calculated using previously established methods by measuring band density with ImageJ software (NIH, Bethesda, Maryland, USA) and normalized to controls (36).

### Flow cytometry.

After 72 hours of incubation with GSK503, the immune cell types were assessed by flow cytometry analyses. GSK503-treated splenocytes and control samples were suspended in 0.5% BSA containing PBS (wash buffer) and were stained with PE anti-mouse CD4 (BioLegend, 116006), FITC anti-mouse CD8a (BioLegend, 100804), APC anti-mouse CD335 (BioLegend, 137608), FITC mouse anti-human CD134 (BD Pharmingen, 555837) and BV650 Mouse Anti-Human CD137 (BD Pharmingen, 564092) antibodies and incubated for 20 minutes on ice. Dead cells were excluded by Sytox Blue staining (Molecular Probes, catalog S34857). Unstained splenocytes were used to detect autofluorescence or background staining. Stained cells were analyzed using a CytoFLEX SRT Flow cytometer (Beckman Coulter) under sterile conditions, and the results were analyzed by FlowJo software version 10.8.1 (Tree Star Inc.).

### RNA-Seq and bioinformatic analysis.

*VCMsh2T*^Hu^ organoid lines were seeded in a 24-well plate at an evenly distributed density and treated with 5 μM GSK503. Untreated organoids with DMSO served as controls. Media was changed after 48 hours to replenish GSK503. On day 5, treated and untreated organoids were collected in cell recovery solution (Corning) and incubated on ice for 1 hour to dissolve residual Matrigel and then pelleted at 390*g* for 5 minutes at 4°C. Total RNA was extracted directly from the pelleted organoids using the Direct-zol RNA MicroPrep kit (Zymo Research, catalog R2062). Similarly, total RNA was also extracted from crypts of control and treated mice. Purified RNA was quantified, and DNAse-I treated (Zymo Research) for bulk RNA-Seq. The libraries from all samples were made and sequenced at the University of Texas MDACC Advanced Technology Genomics Core (ATGC) on the Illumina Hi-Seq4000 instrument generating paired-end 76 bp reads. Transcript reads were aligned to the GRCm39 mouse genome assembly using STAR (v2.7.9a) (37). Counts were calculated from the resulting bam files using rsem (v1.3.1) (38). Differential gene expression analyses were performed using EdgeR (v3.123) (39) with TMM normalization. Genes with a Benjamini-Hochberg–adjusted *P* < 0.05 and log_2_FC < 1 were considered significant and selected for the Volcano plot. GSEA was conducted using clusterProfiler (v3.0.45) (40) with the rank of “Signed fold change * –log_10_(*P* value).” Additionally, in silico deconvolution of immune cell types was performed using the BASE (41) algorithm implemented by the R package immueconv v2.1.1 (41).

### Single-cell RNA-Seq analysis.

Mouse colons were aseptically removed, flushed with PBS, and cut into 2–3 cm pieces. The tissue was washed with PBS and incubated in 1 mg/mL Collagenase IV (Thermo Fisher Scientific, catalog 17104019) in Advanced DMEM/F12 HEPES with penicillin/streptomycin at 37°C for 1 hour. The mixture was homogenized using a glass dounce homogenizer and filtered through 70 μm (Thermo Fisher Scientific, catalog 22363547) and 40 μm (Thermo Fisher Scientific, catalog 22363548) cell strainers. Cells were washed with ice-cold PBS, centrifuged at 600*g* for 5 minutes, and resuspended in cell staining media for further analysis. Isolated single cells from murine colon were processed using the 10X Genomics Chromium Next GEM Single Cell 3′ V2 chemistry following the manufacturer’s protocol (CG000204). Briefly, cells were loaded onto a Chromium Next GEM Chip K (10X Genomics PN, 1000286) at the recommended concentration to capture between 7,000 and 10,000 cells along with Single Cell 3′ Gel Beads containing barcoded oligos. Using the Chromium Controller, thousands of cells were partitioned into nanoliter-scale Gel Bead-in-Emulsion (GEM) droplets for single-cell reactions. Within the droplets, cells were lysed, and the cellular RNA was barcoded using reverse transcriptase from the poly(dT) room temperature primers, resulting in full-length cDNA from polyadenylated mRNA. The barcoded RNA was then released, pooled, and amplified to construct the 3′ RNA libraries using the Chromium Next GEM Single Cell 3′ Reagent Kit v2 (10X Genomics PN, 1000268).

### ChIP-Seq analysis.

Crypts from *VCMsh2T^Hu^* mice were pooled from treated and control animals (*n* = 5 per group). Samples were submitted to the MDACC Epigenomics Core for chromatin immunoprecipitation using antibodies specific for H3K27me3, H3K4me3, H3K4me1, and H3K27ac. Immunoprecipitated DNA from each sample was used for library preparation and subsequent sequencing. For all ChIP-Seq analyses, reads were mapped to the GRCm39 mouse genome using BWA-MEM 0.7.11 (arXiv:1303.3997). Duplicate reads were identified using the GATK (v4.2.3.0) package (42). Samtools (v1.14) (43) was used to filter unmapped, duplicated, and low-mapping-quality sequences (MAPQ <30). MACS2 (v2.1.2) (44) was used for peak calling with the default settings. For H3K4me3 and H3K4me1 peak calling, broad peak mode was used, otherwise narrow peak mode was used. Differential peaks in control and treatment samples were identified by MACS2. The heatmap of ChIP-Seq signal around TSS or peaks was plotted using deeptools (v3.1.3) (45). The peaks were annotated using HOMER (v4.11) (46) for functional analysis and viewed in IGV (v2.16.0) (47).

### Statistics.

Comparisons between two experimental groups were performed with GraphPad Prism (RRID:SCR_002798) using 2-tailed Student’s unpaired *t* test. For parametric data, comparisons between more than two experimental groups were analyzed using 1-way ANOVA with Tukey’s post hoc analysis for multiple comparisons. For nonparametric data, comparisons between more than two experimental groups were analyzed using Kruskal-Wallis test with multiple comparisons. The data are expressed as mean ± SEM from technical triplicates of 3 biological replicates. *P* values of less than 0.05 were considered significant.

### Study approval.

All patient samples and information were collected with written informed consent in accordance with the MDACC IRB protocol and US Common Rule regulations. The study conformed to the guidelines from the IRB-approved protocol (MDACC IRB, no. PA12–0327). Tissue specimens were obtained during routine screening and diagnostic procedures from patients followed at the University of Texas MDACC. All animal experiments were approved by the IACUC of the University of Texas MDACC (MDACC), and the care of the animals was in accordance with institutional guidelines (IACUC protocol no. 00000469-RN02).All animal experiments were conducted in compliance with the NIH guidelines for animal research.

### Availability of data and materials.

Datasets generated and analyzed in the current study can be accessed for reanalysis in Supplemental Tables 1–11. Organoid data may be reviewed by accessing the GEO database (GSE277544). Tissue data may be reviewed by accessing GEO database (GSE277940) Values for all data points in graphs are reported in the Supporting Data Values file.

## Author contributions

EV has full access to all the data in the study and takes responsibility for the integrity and accuracy of the data analyses. EV conceived and supervised the study, provided critical resources to perform the experiments, and wrote the manuscript. CMB, FD, AMM, LRU, MZ, SM, RS, and KMS performed the experiments, analyzed data, and wrote the manuscript. ND performed the bioinformatics analyses. JT managed the mouse colony and provided assistance during experiments. MWT evaluated all histological samples and provided clinical expertise in pathology. AKJ provided assistance and technical support for ChIP-Seq experiments. WE provided assistance on the analysis and interpretation of the data as well as writing and editorial assistance. EV provided identification of study participants and clinical information. ET generated the VCMsh2THu mouse line used in this study. All authors critically read and intellectually contributed to the manuscript. Order for co–first authors was decided based on level of contribution to the work herein.

## Supplementary Material

Supplemental data

Unedited blot and gel images

Supplemental tables 1-11

Supporting data values

## Figures and Tables

**Table 1:**
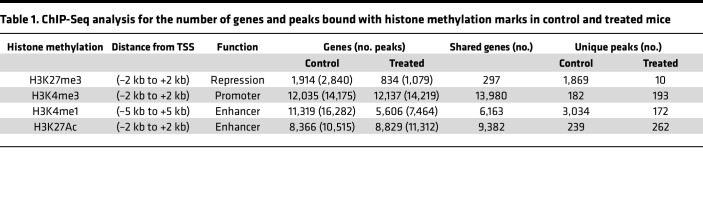
ChIP-Seq analysis for the number of genes and peaks bound with histone methylation marks in control and treated mice

## Version 1 02/13/2025

In-Press Preview

## Version 2 03/24/2025

Electronic publication
