# Supplementary material for: Inhibition of histone methyltransferase EZH2 for immune interception of colorectal cancer in Lynch syndrome
Source: JCI Insight. 2025 Feb 13;10(6):e177545. doi: 10.1172/jci.insight.177545 (PMC11949072; doi:10.1172/jci.insight.177545)

Two-dimensional cell culture

Human colorectal cancer cell lines HCT116 (microsatellite unstable) and SW620 (microsatellite stable) [ATCC CCL-24 and CCL-227] were grown in DMEM with 4.5 g/L glucose (Mediatech) supplemented with 5% FBS (Sigma-Aldrich), and 1% penicillin/streptomycin (HyClone). Cells were incubated at 37°C with 5% CO2. Upon acquisition, all cell lines were authenticated by the Cytogenetics and Cell Authentication core at MDACC and are regularly tested for mycoplasma contamination.

Lentiviral Transduction of Organoids

To knock down Ezh2 in LS mouse-derived organoids, cells were transduced with a lentivirus encoding three different short-hairpin RNAs against Ezh2 (Ezh2_sh) under the control of the miR30 promoter. To transduce organoids with lentivirus, cells were first washed with PBS and then dissociated into smaller clusters using TrypLE^{TM} Express (Gibco) for 5 minutes at 37°C. Then, organoids were resuspended in 500 µl of transduction mix containing 10 µM Y-27632, 8 µg/mL polybrene, and concentrated lentivirus in organoid culture media. The resuspended organoids were centrifugated at 600 × g for 30 minutes at 20°C. After centrifugation, the organoids were incubated overnight at 37°C and then replated into a new 24-well plate. The pLV-mEzh2[miR30] construct includes a puromycin-resistance gene, so puromycin was used for selection in organoid cultures.

Human organoid and peripheral blood mononuclear cell co-culture experiment.

Organoids were established from patient-derived colon tumor biopsies. Tissues were minced and digested using Gentle Cell Dissociation Reagent (GCDR, Stem Cell Technologies) to create a

single-cell suspension. The cell suspension was centrifuged and resuspended in Matrigel, then plated in small domes in a pre-warmed culture plate. The domes were overlaid with IntestiCult™ Organoid Growth Medium (Human) supplemented with Organoid Supplement (Stem Cell Technologies) and incubated at 37°C with 5% CO2.

Peripheral blood mononuclear cells (PBMCs) were isolated from patient blood samples using Corning® LSM (Lymphocyte Separation Medium). The isolated PBMCs were then washed and resuspended in RPMI-1640 medium supplemented with 10% fetal bovine serum (FBS) and 1% penicillin-streptomycin. For the co-culture setup, 100,000 PBMCs were seeded into each well of a 24-well plate, which already contained organoids embedded in Matrigel domes. The co-cultures were maintained at a 1:1 effector (PBMCs) to target (organoids) ratio in a 1:1 mixture of IntestiCult™ Organoid Growth Medium (with Organoid Supplement) and RPMI-1640 medium supplemented with 10% FBS and 1% penicillin-streptomycin. The cultures were incubated at 37°C with 5% CO2 for 24 hour. The medium was supplemented with three different doses of GSK503 (0.5 μM, 1 μM, and 2 μM). Media without the drug served as a control. Parallel experiments were conducted with organoids alone treated with the same doses of GSK503.

Fully automated 22-plex sequential IF panels on FFPE tissue sections. The seqIF approach on COMET™ was used to develop a 22-plex panel on FFPE mouse colon tissue. The panel consisted of biomarkers for immune regulators, epithelium, and stroma: CD3e, CD8, CD4, Ki67, PanCK, COL1A1, B220, LY6C, Foxp3, TCF7, CD68, LGR5, F4/80, Granzyme B, CD163, Cleaved caspase 3, E-Cadherin, SPP1, CD11b, CD31, TBET and αSMA (**Table S2**). Prior to sequential immunofluorescence (seqIF), FFPE tissue slides were deparaffinized in xylene followed by

rehydration in a graded alcohol series and blocked with 3% hydrogen peroxide for 10 min. Antigen retrieval was performed with EZ-AR2 Elegance buffer (BioGenex, Fremont, CA, USA) at 107 °C in an EZ-Retriever system V.3 (BioGenex, Fremont, CA, USA) for 15 min. The processed slides were then transferred to a Multistaining Buffer (BU06, Lunaphore, Tolochenaz, Switzerland) bath until use [1]. The microfluidic chip (9 × 9 mm imageable area) was clamped against the FFPE tissue section on a standard microscope slide forming a closed reaction chamber. The reagents were delivered through microfluidic channels under highly controlled conditions. Slides underwent 11 cycles of iterative staining and imaging, followed by an elution of the primary and secondary antibodies on COMET™ platform (Lunaphore Technologies)[1, 2]. The 22-plex protocol template was generated using the COMET™ Control Software, and reagents were loaded onto the device to perform the sequential immunofluorescence (seqIF™) protocol. Images were taken by the integrated epifluorescent microscope at 20× magnification using DAPI (exposure time, 80 ms), TRITC (exposure time, 400 ms), and Cy5 (exposure time, 200 ms) channels for every cycle with an imaging area of 9 × 9 mm. List of primary antibodies with corresponding incubation times is enclosed in **Table S2.** Secondary antibodies were used as a mix of 2 species-complementary antibodies plus DAPI, [Alexa Fluor Plus 647 goat anti-mouse (Thermo Scientific, cat no: A32728, 1/200 dilution)] and [Alexa Fluor Plus 555 goat anti-rabbit (Thermo Scientific, cat no: A32732, 1/100 dilution)] or [Alexa Fluor Plus 647 goat anti-rabbit (Thermo Scientific, cat no: A32733, 1/200 dilution)] and [Alexa Fluor Plus 555 goat anti-mouse (Thermo Scientific, cat no: A32727, 1/100 dilution)]. The seqIF™ protocol in COMET™ resulted in a multi-layer OME-TIFF file where the imaging outputs from each cycle are stitched and aligned. COMET™ ome.tiff contains DAPI image, intrinsic tissue autofluorescence in TRITC and Cy5 channels, a single fluorescent layer per marker and single layer per additional image post-elution.

Image analysis was conducted using Visiopharm image analysis software version 2023.09 x64 (Visiopharm Inc., Hoersholm, Denmark). Fluorescent images of layers 1, 2, and 3 for each sample were first aligned to the corresponding MSI images using the Tissuealign module, resulting in a 3-dimensional image. The seqIF layer was utilized for tissue segmentation to distinguish between different tissue regions. Tissue segmentation was performed utilizing pan-keratin and αSMA markers to define epithelium, stroma, and muscle regions, respectively. After tissue segmentation, cell boundaries were determined by a pretrained machine learning algorithm that used DAPI channel to automatically identify nuclei and cells. Identified cells were then phenotyped using Visiopharm's unbiased autoclustering module using only the top 20% of pixel values per cell.

References

1. Rivest, F., et al., *Fully automated sequential immunofluorescence (seqIF) for hyperplex spatial proteomics.* Sci Rep, 2023. **13**(1): p. 16994.
2. Migliozzi, D., et al., *Microfluidics-assisted multiplexed biomarker detection for in situ mapping of immune cells in tumor sections.* Microsyst Nanoeng, 2019. **5**: p. 59.

Supplemental Figure 1. A. Principal component analysis for AP and NM organoids with and without GSK503 treatment. Blue color represents normal tissue (NM); red color represents polyp tissue (AP); circular dots correspond to GSK503 treated samples; squares correspond to control samples; **B.** Heatmap using a cut-off of LogFC>0.5 (FDR<0.05) for AP and NM organoids showing a significant difference in differentially expressed genes (~1600 genes) in treated AP-MDOs compared to untreated AP-MDOs.

Supplemental Figure 2. Gene Set Enrichment Analysis highlighting activated (**A**, left) and suppressed (**B**, right) pathways as determined from RNAseq data generated from *VCMsh2*^{Hu} AP and NM organoids.

Supplemental Figure 3. A. Dose-response curve assessing the viability of mouse-derived organoid (MDO) upon treatment with EZH2 inhibitors, GSK-503 and tazemetostat; **B.** The representative well image of MDO treated with inhibitors and DMSO from panel A. Calculated IC_{50} for organoids treated with GSK503 and tazemetostat show 16.08 μM and 19.19 μM, respectively; **C.** Dose-response curves for GSKS503 and tazemotostat using colorectal cancer cell lines, HCT116 (MSI, microsatellite instable) and SW620 (MSS, microsatellite stable); **D.** Western blot showing decreased H3K27me3 levels upon serial dosing of GSK503 in MDOs for 5 days. The graphed data are expressed as means ± SEM. For all graphs, One-way ANOVA with multiple comparisons was used to determine significance; (*, P<0.05; **, P<0.01; ***, P<0.001; ****, P<0.0001).

Supplemental Figure 4. Flow cytometry gating strategy utilized for immune profiling and organoid co-culture experiments. All gates and interpretations were established by personnel in the MDACC Flow Cytometry Core Facility.

Supplemental Figure 5. Murine body weight from pre-clinical trial. No significant difference in body weight was determined at any point in the trial as determined using multiple Student's t-test.

Supplemental Figure 6. A. Representative merged images from Comet analysis of control and treated mouse colon tissues (N=3 mice/group). Channel colors correspond to respective marker denoted below each image; **B.** Quantification analysis of Comet data from *VCMsh2T*^{hu} mice with significance determined using Student's t-test.

Supplemental Figure 7. A. Western blot shows the efficiency of Ezh2-knockdown by mouse-specific Ezh2 using lenti-shRNA in murine organoids from *VCMsh2T*^{hu} mice. MDOs transduced with lenti-scrambled shRNA was used as control. The protein expression of each marker was normalized to its corresponding β-actin. Histone H3 was used to normalize H3K27me3 levels. A total of 20 μg of cell lysate per sample was used to study the levels of Ezh2, histone H3, and H3K27me3. For the analysis of Lgr5, 100 μg of protein lysate was used for each sample; **B.** Green-fluorescent protein expression shows efficiency of transduction of lenti-shRNA construct in organoids. Images are shown at 4X magnification.

Supplemental Figure 8. A. Results from *in silico* deconvolution of immune cell types performed using the BASE algorithm on data obtained from RNA sequencing of murine colonic crypts from GSK503 pre-clinical trial; **B.** Dot plot shows the enrichment of activated (top) and suppressed (bottom)WikiPathways gene sets in treated mice compared to controls. The size of each dot represents the number of core enrichment (leading-edge) genes in the gene set. The color of each dot represents the *P* for the enrichment. The gene sets are ordered by the gene ratio, which is the number of significant genes associated with the gene set divided by the total number of significant genes; **C.** A heatmap of gene expression for each cell cluster generated from single-cell RNA

sequencing analyses. Colored bar above heatmap corresponds to cell cluster identity as described in the color-coded legend.

Supplemental Figure 9. An IGV snapshot of the representative histone methylation levels of H3K4me1, H3K27ac and H3K4me3 at 1.6mb genomic fragment of the chromosome 6 in the crypts from control and treated mice.

Supplemental Figure 10. Analysis of EZH2-target genes. **A.** scRNA-seq and RNA-seq identified 9,964 and 5,862 upregulated genes (LogFC>0.1), respectively, with 3,105 genes common to both datasets. From 1,103 genes with low H3K27me occupancy (ChIP seq analysis) in GSK503-treated mice, 290 were upregulated in scRNA-seq **(B)** and 225 in RNA-seq **(C),** suggesting direct control of EZH2 over these genes via histone H3K27 methylation.

Supplemental Figure 11 A. RNA expression levels of EZH2 were assessed in a cohort of LS patients at MDACC, with results plotted as Log_{10} counts per million (CPM). Statistical analysis was conducted using a Kruskal-Wallis test with multiple comparisons; **B.** Immunohistochemistry results of EZH2 staining in human colorectal tissue from LS patients. Results were scored by an expert pathologist using the H-scoring method and graphed as H-score of EZH2 (y-axis). Gray scale bar = 200 μm. Nm=normal; AdCA= adenocarcinoma; Dysplasia= AdCA+Adenoma. Statistical analysis was conducted using a One-way Anova for dot-plot and Student's t-test for box-plot.

Supplemental Table 1. RNAseq (count per million) analysis in normal mucosa organoid (NM) and adenomatous polyp organoid (AP) of LS *VCMsh2*^{Hu} mice in the presence or absence of GSK503 treatment.

Supplemental Table 2. Markers stained for the 22-plex Lunaphore COMET panel and their associated cell phenotypes.

Supplemental Table 3. RNAseq analysis in colonic crypts of GSK503-treated and control *VCMsh2*^{Hu} mice.

Supplemental Table 4. ChIPseq analysis of H3K27me3 in control *VCMsh2*^{Hu} mice.

Supplemental Table 5. ChIPseq analysis of H3K27me3 in GSK503-treated *VCMsh2*^{Hu} mice.

Supplemental Table 6. ChIPseq analysis of H3K4me3 in control *VCMsh2*^{Hu} mice.

Supplemental Table 7. ChIPseq analysis of H3K4me3 in GSK503-treated *VCMsh2*^{Hu} mice

Supplemental Table 8. ChIPseq analysis of H3K4me1 in control *VCMsh2*^{Hu} mice.

Supplemental Table 9. ChIPseq analysis of H3K4me1 in GSK503-treated *VCMsh2*^{Hu} mice.

Supplemental Table 10. ChIPseq analysis of H3K27ac in control *VCMsh2*^{Hu} mice.

Supplemental Table 11. ChIPseq analysis of H3K27ac in GSK503-treated *VCMsh2*^{Hu} mice.

Supplemental Figure S1

A.

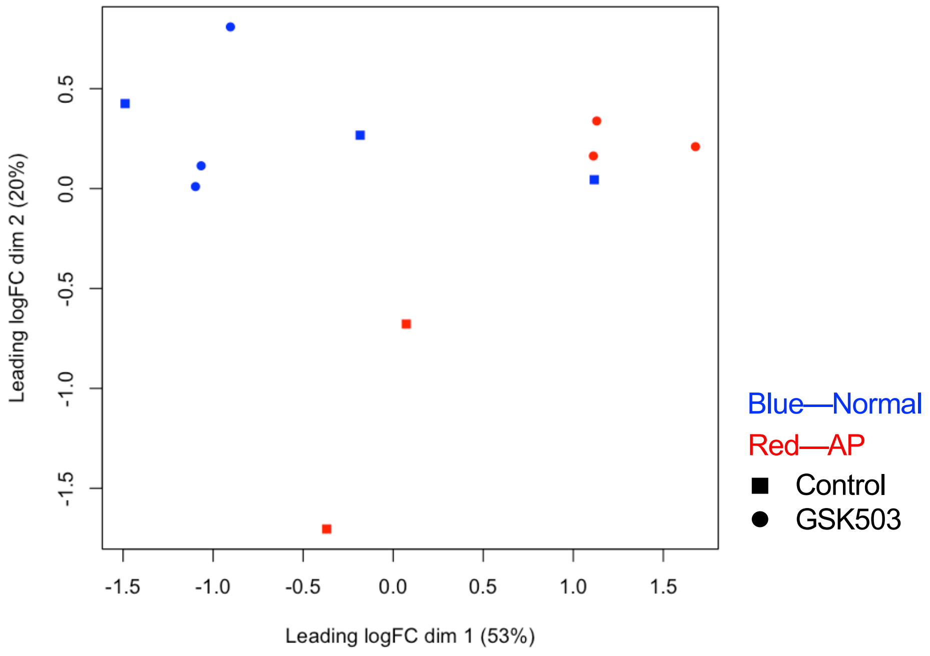

B.

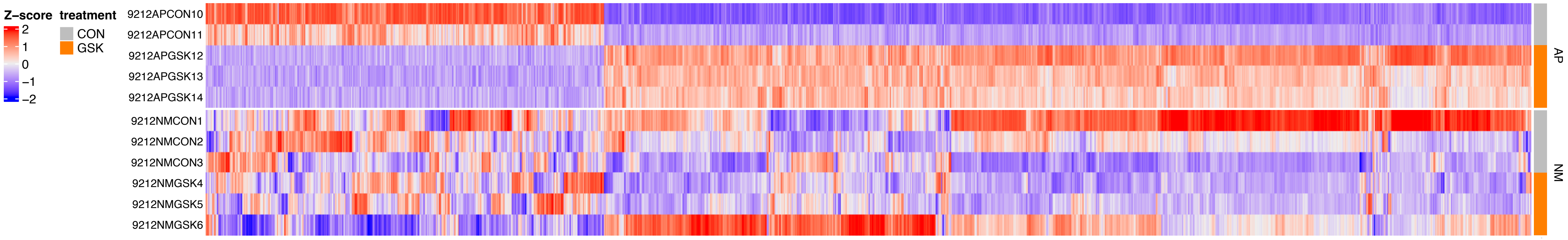

Supplemental Figure S2

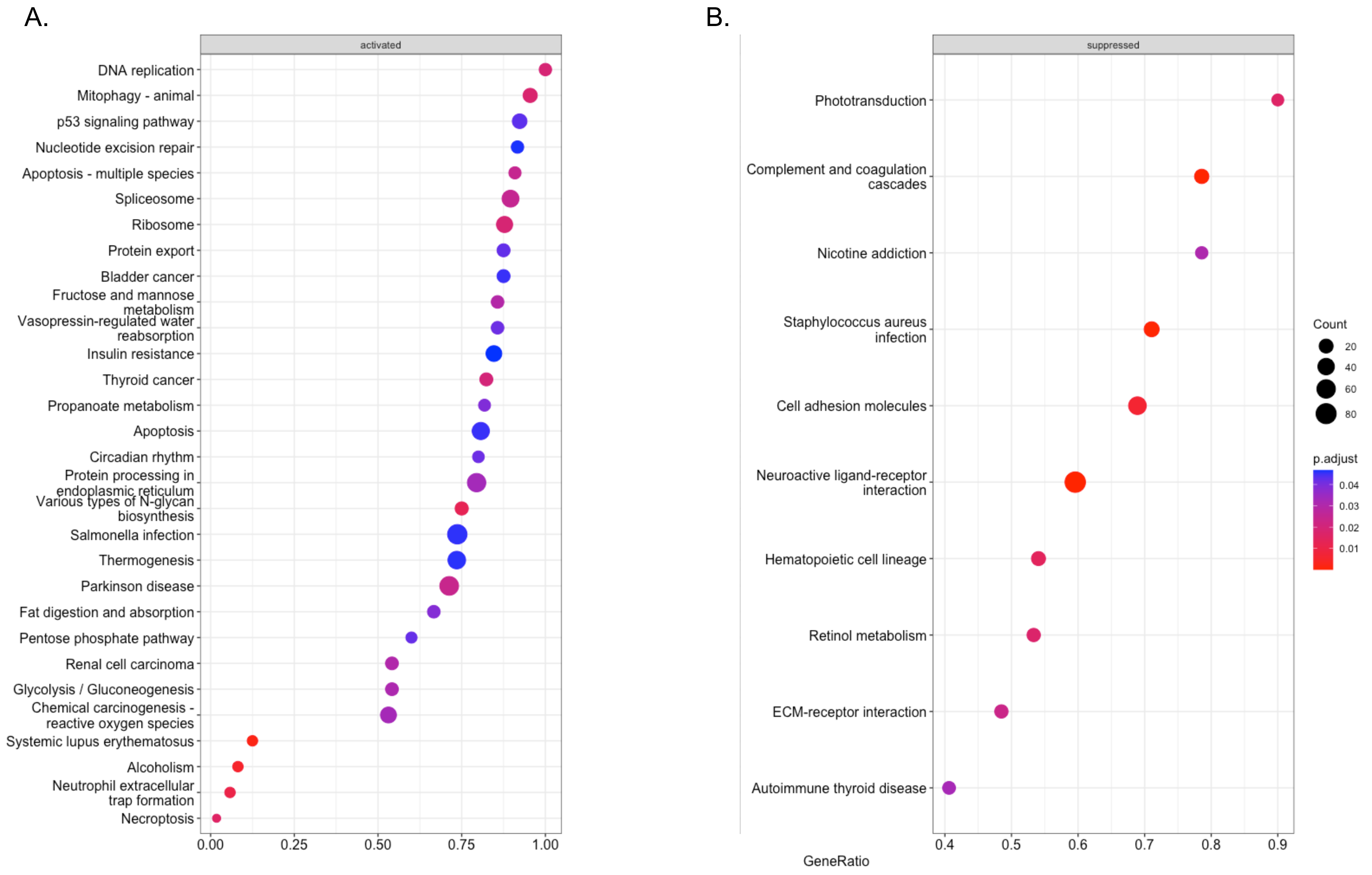

Supplemental Figure S3

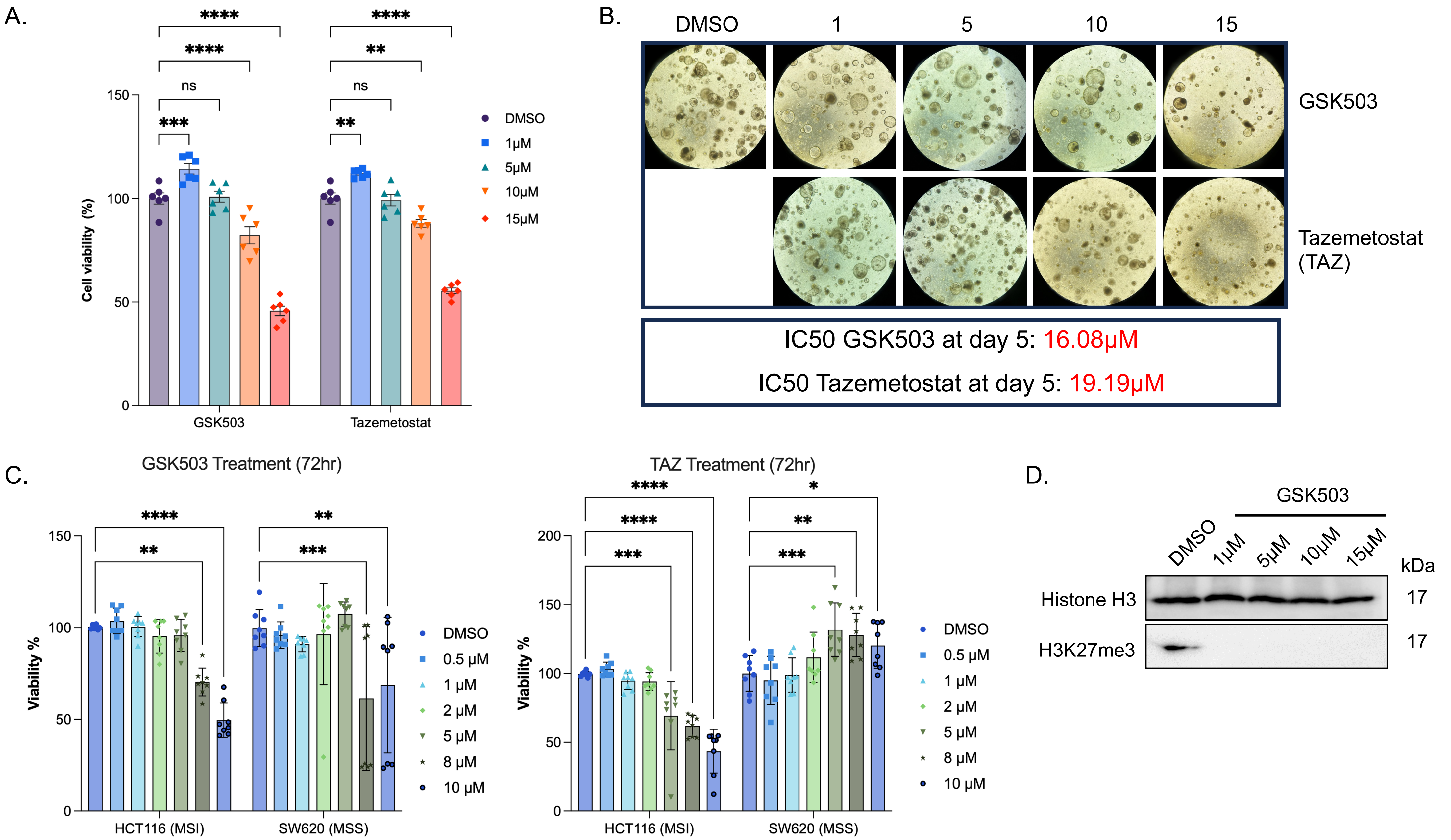

Supplemental Figure S4

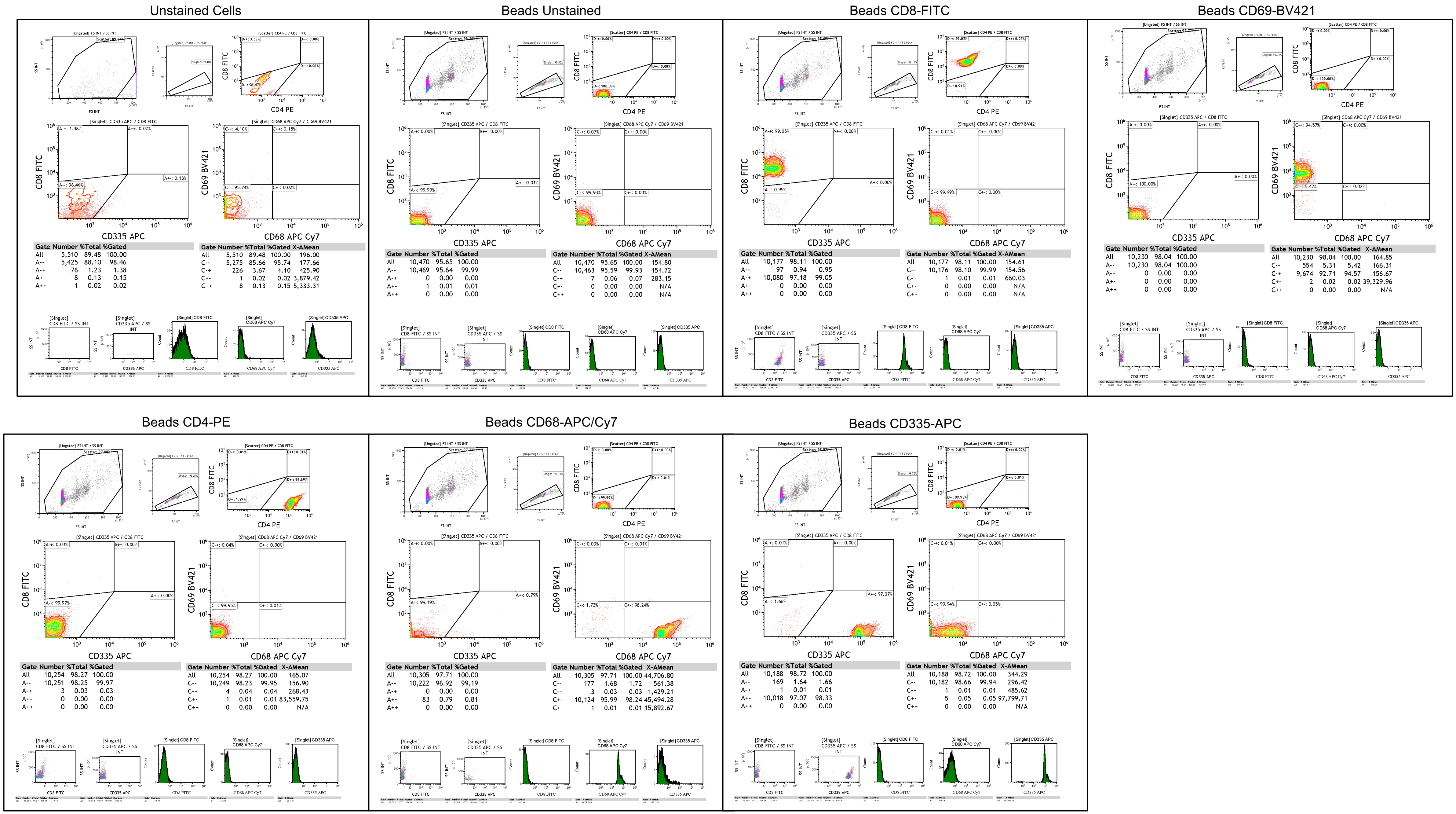

Supplemental Figure S5

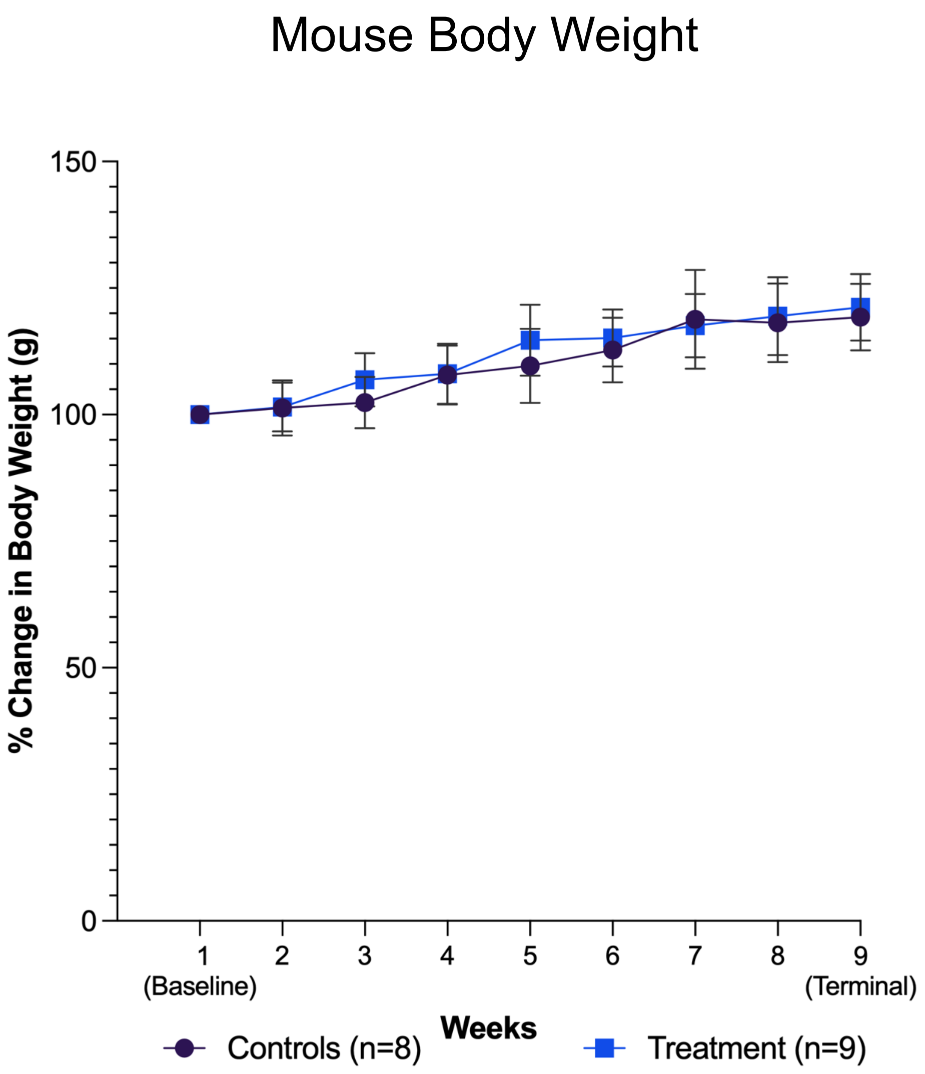

Supplemental Figure S6

A

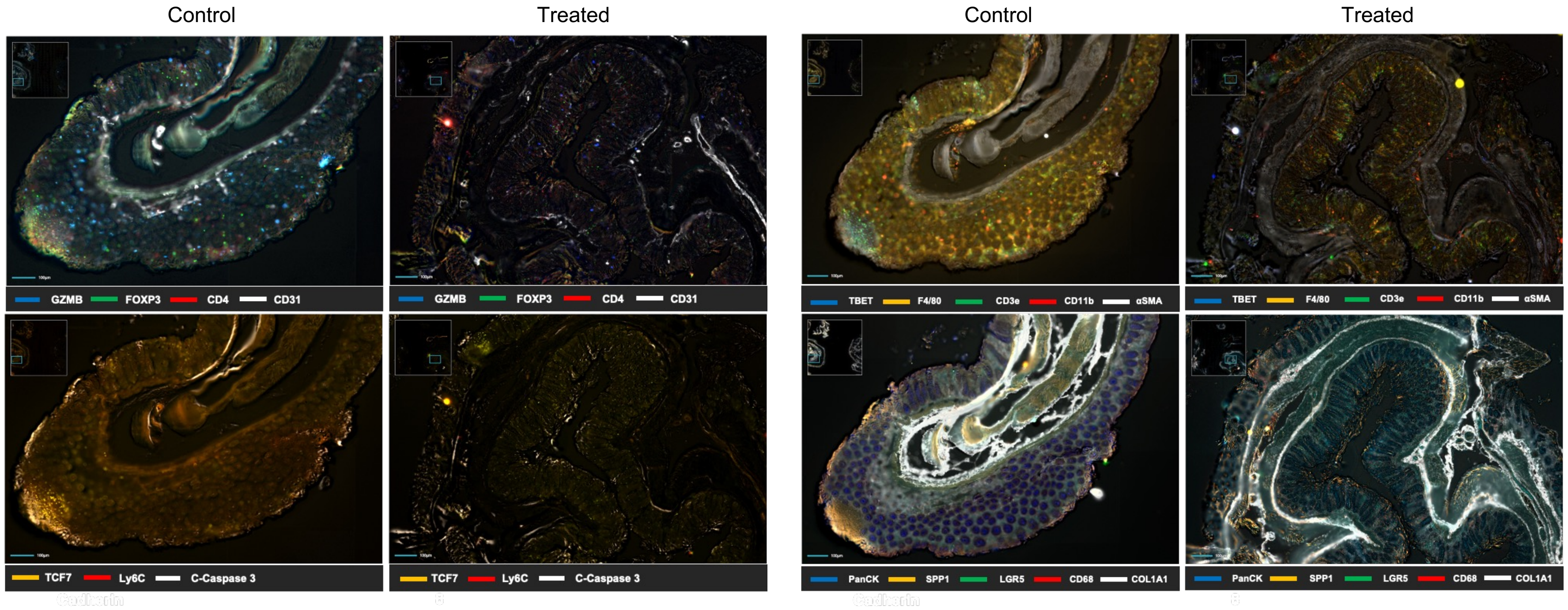

B

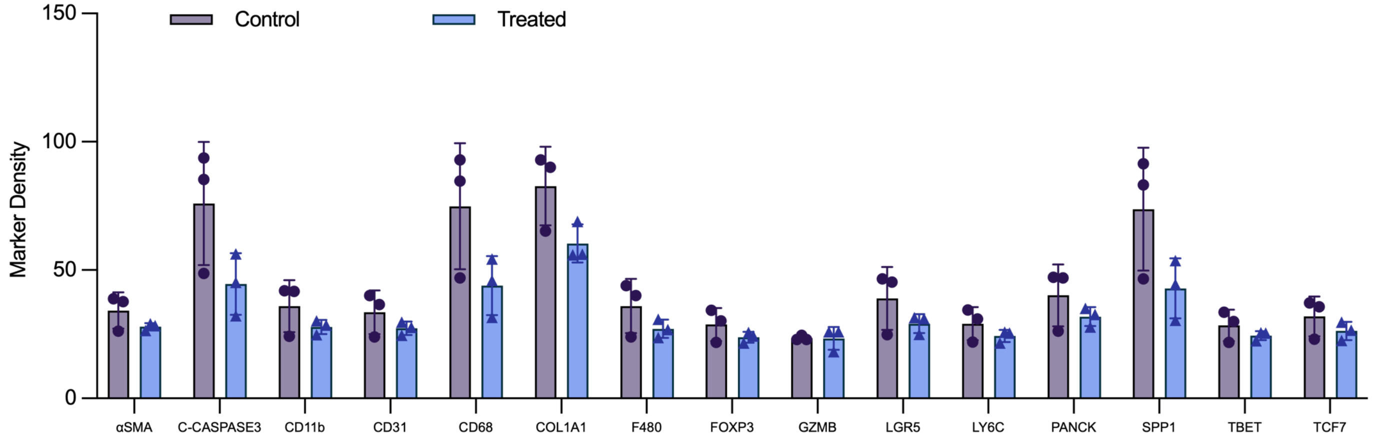

Supplemental Figure S7

A.

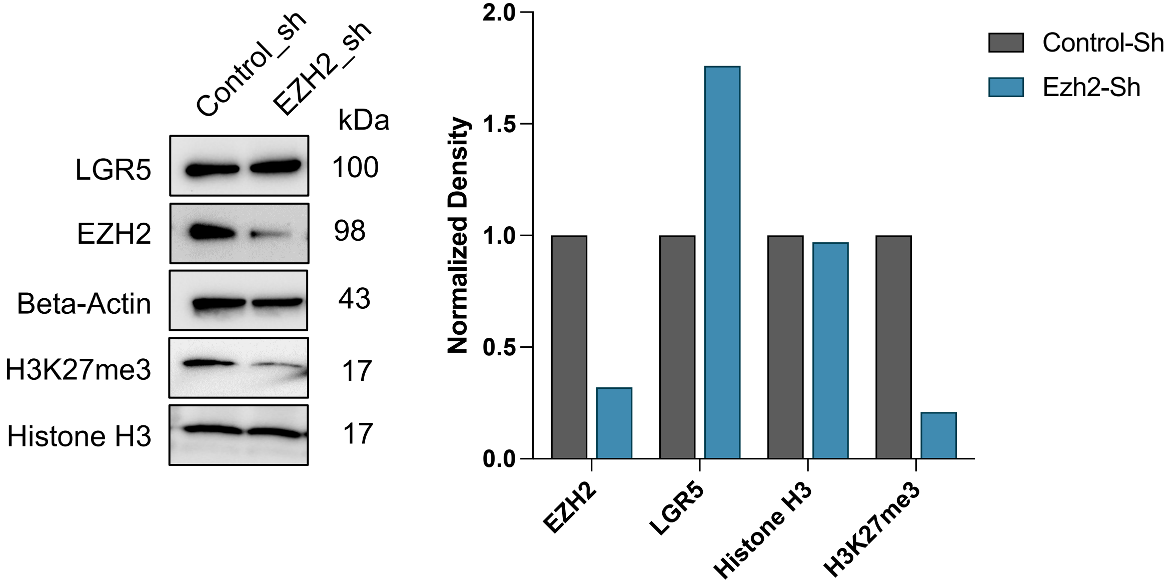

B.

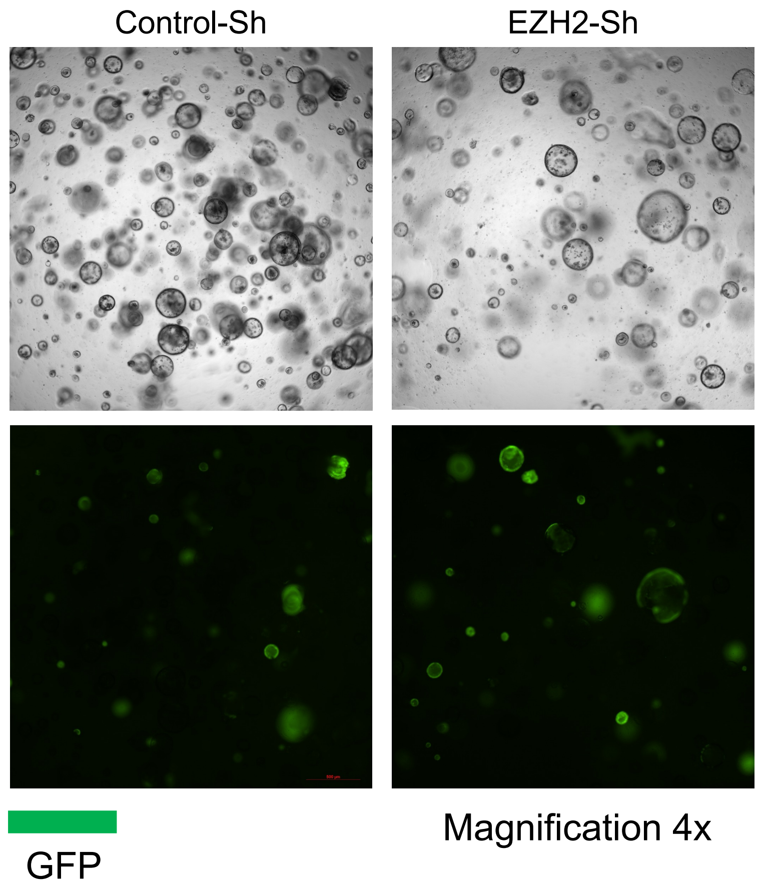

Supplemental Figure S8

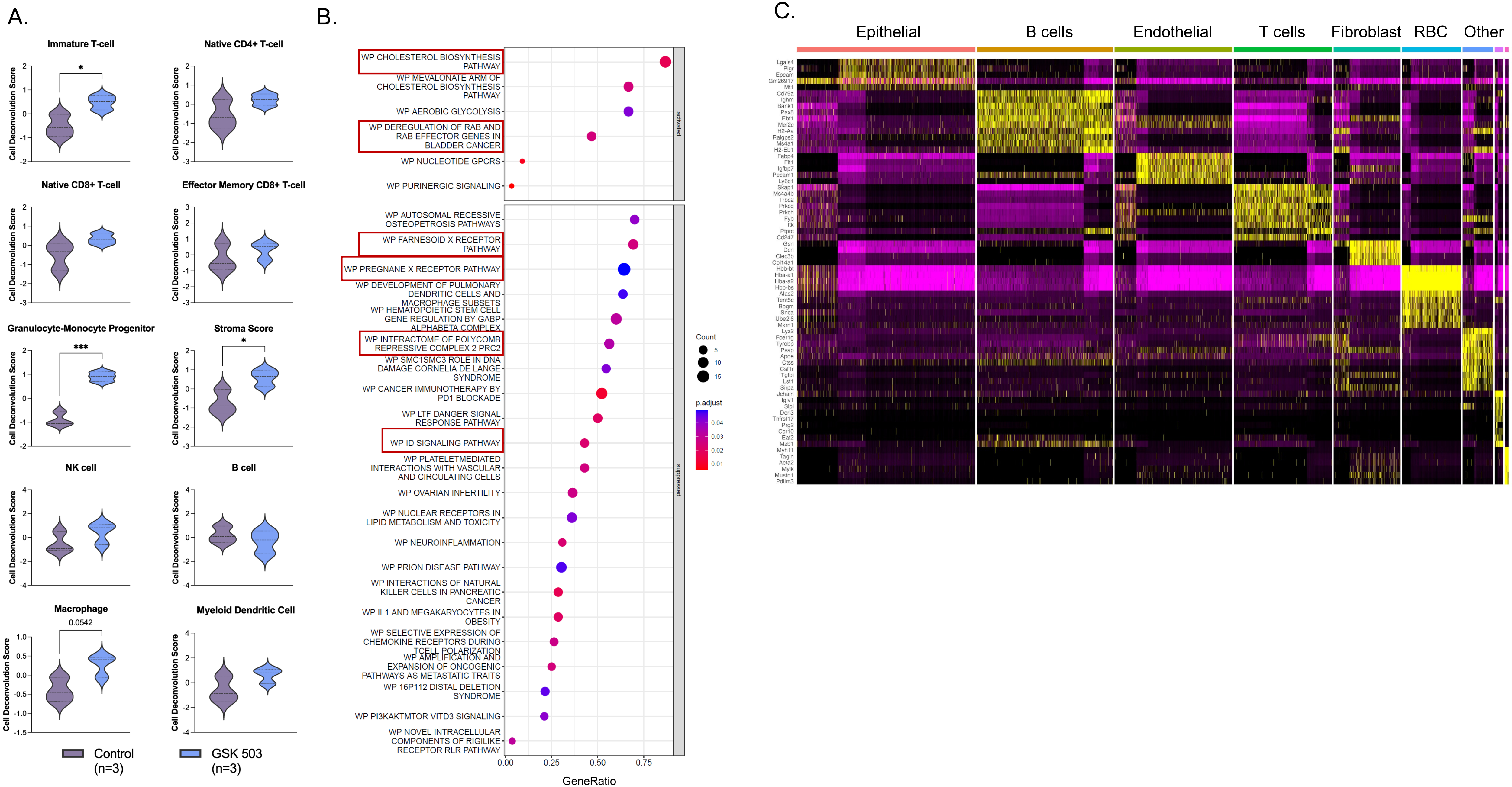

Supplemental Figure S9

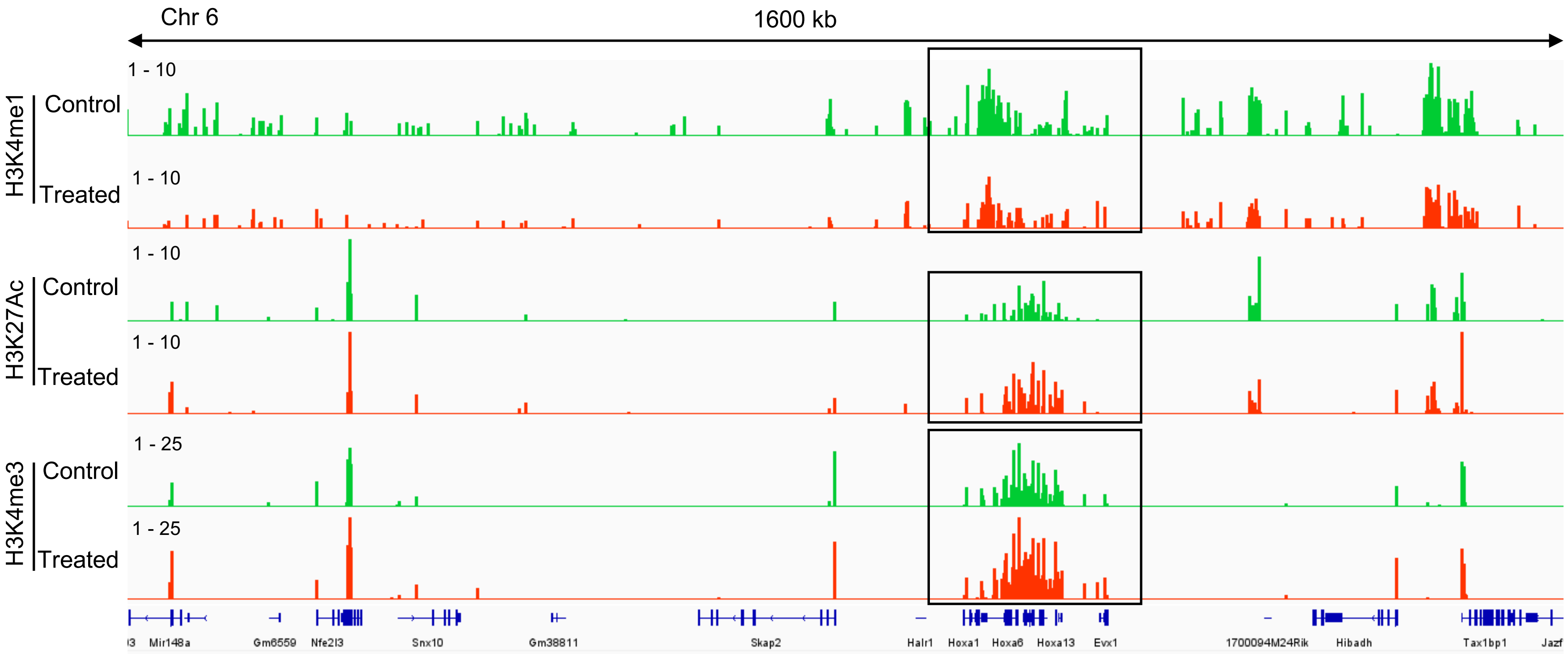

Supplemental Figure S10

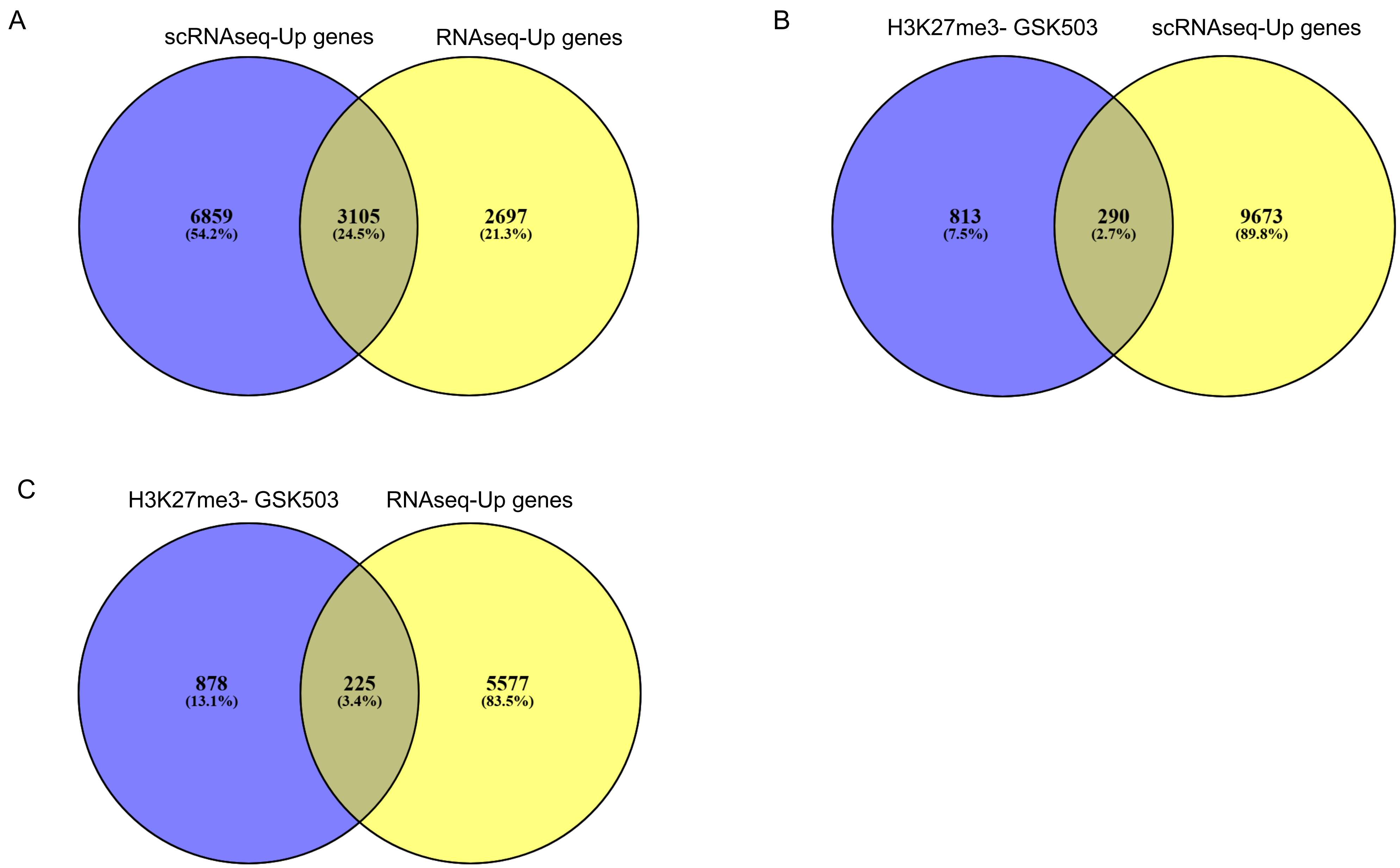

Supplemental Figure S11

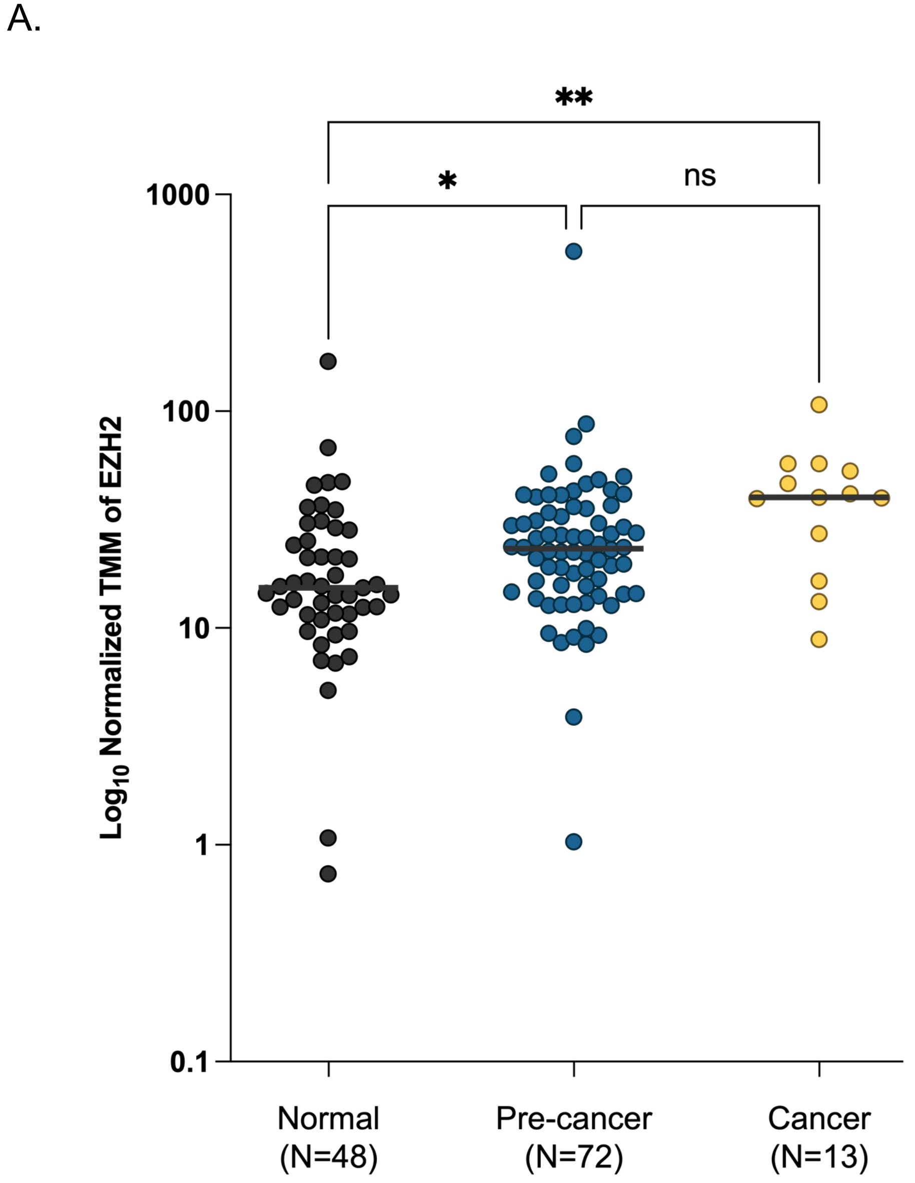

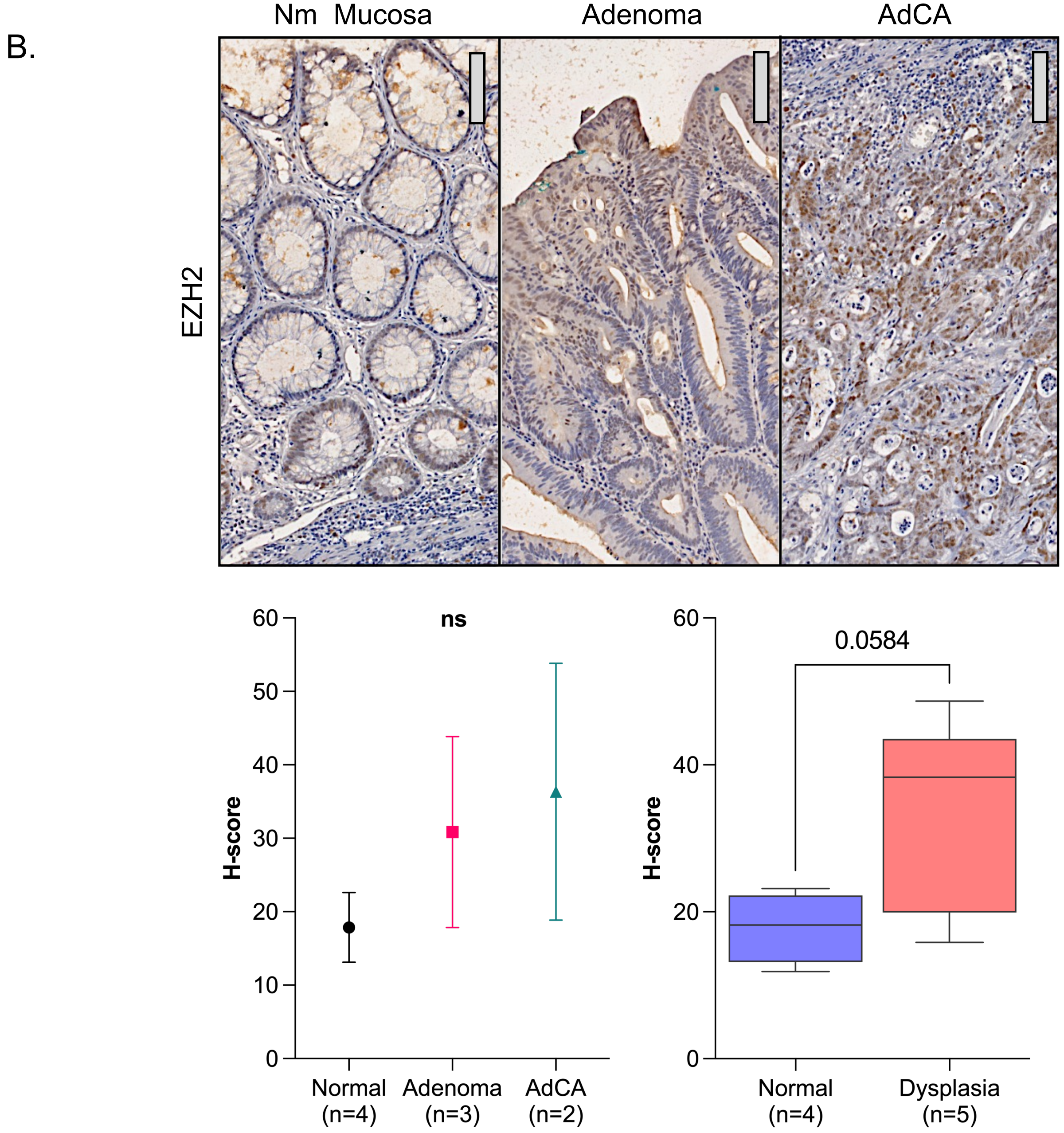

Supplement: Supplemental data [file jciinsight-10-177545-s282.pdf]
