# Supplementary material for: Inhibition of histone methyltransferase EZH2 for immune interception of colorectal cancer in Lynch syndrome
Source: JCI Insight. 2025 Feb 13;10(6):e177545. doi: 10.1172/jci.insight.177545 (PMC11949072; doi:10.1172/jci.insight.177545)

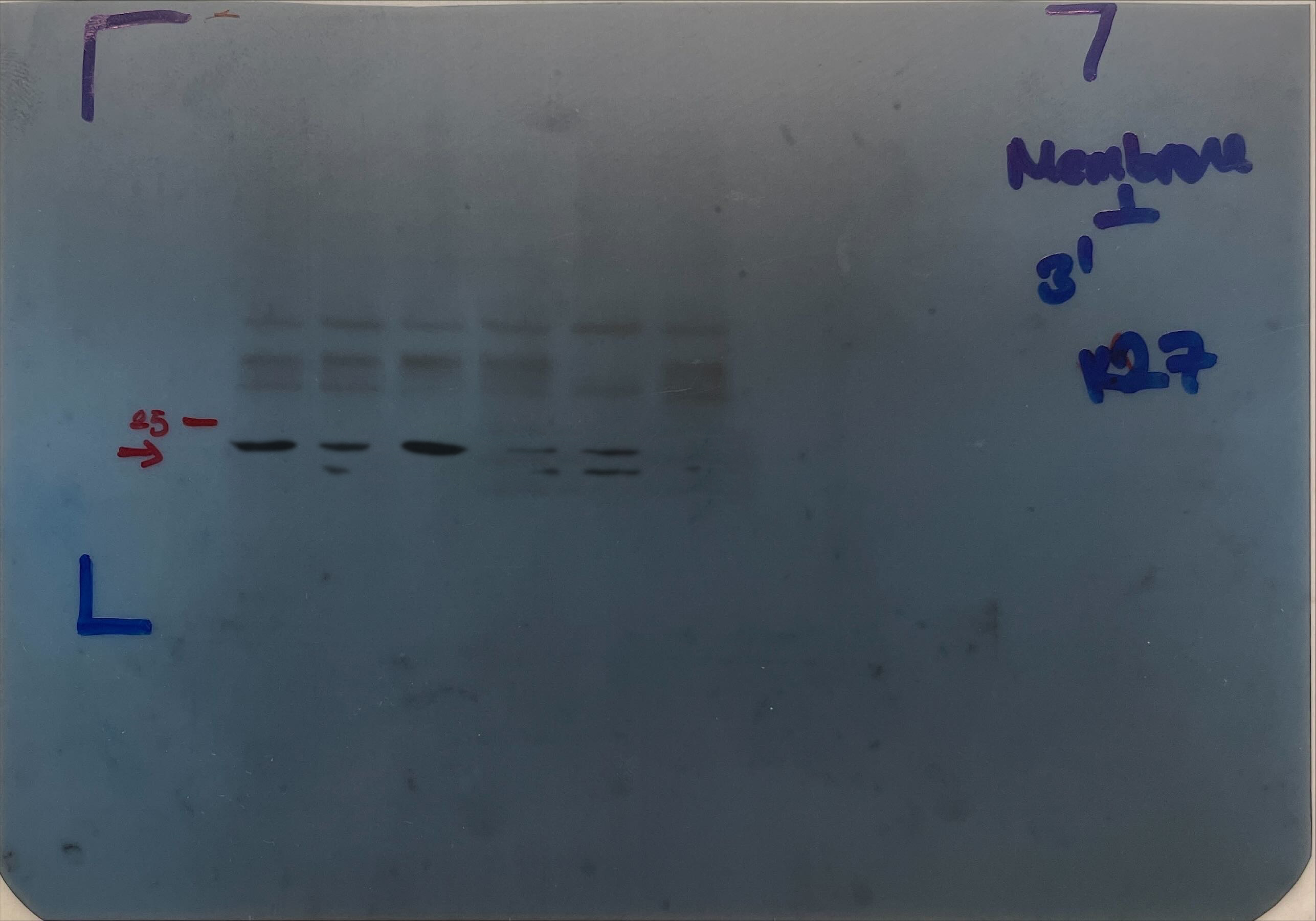
85 –
Membrane
1
3'
K27

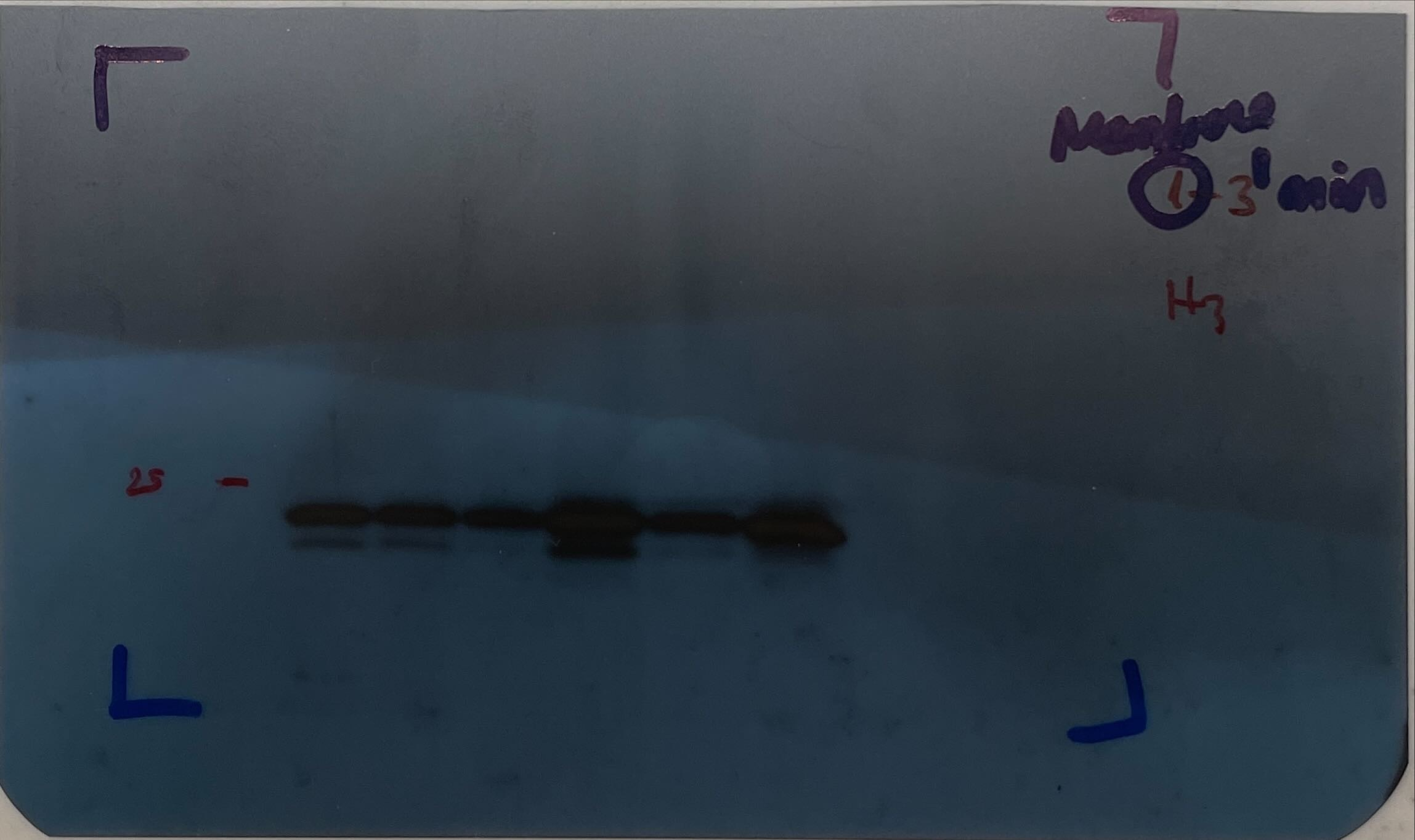

Membrane
1 - 3' min
H3
25

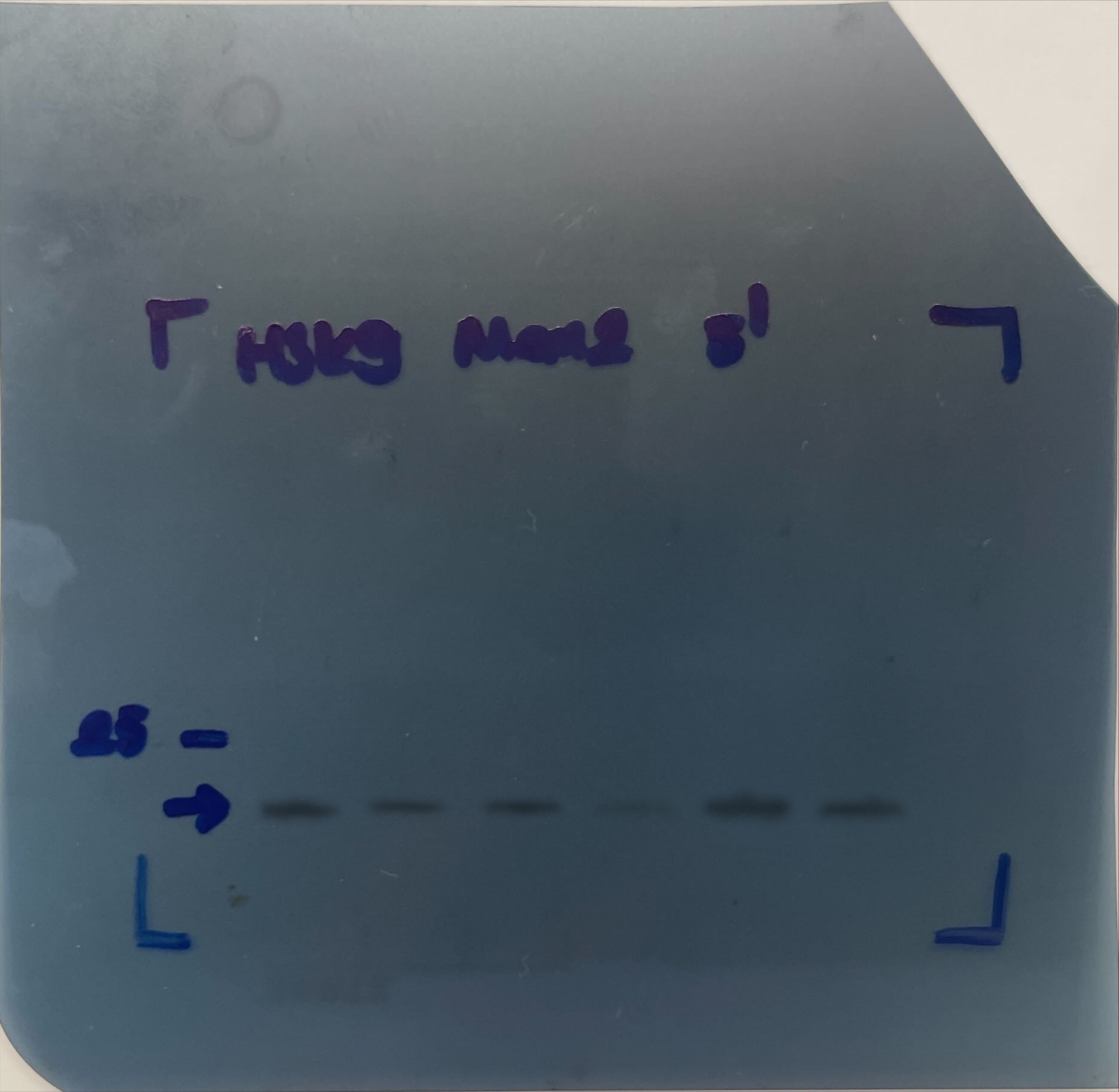
H3K9 Mem2 5'
25

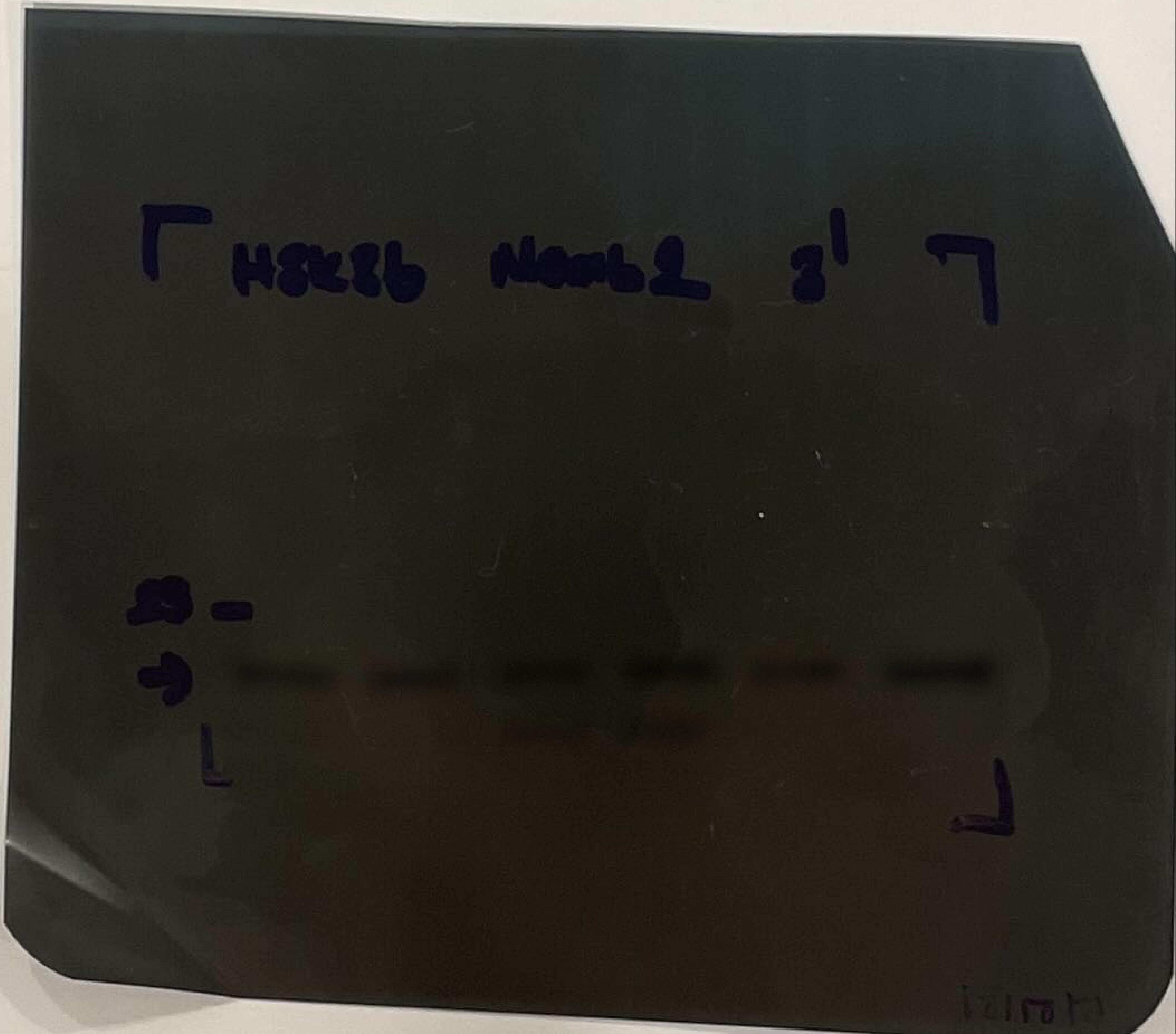

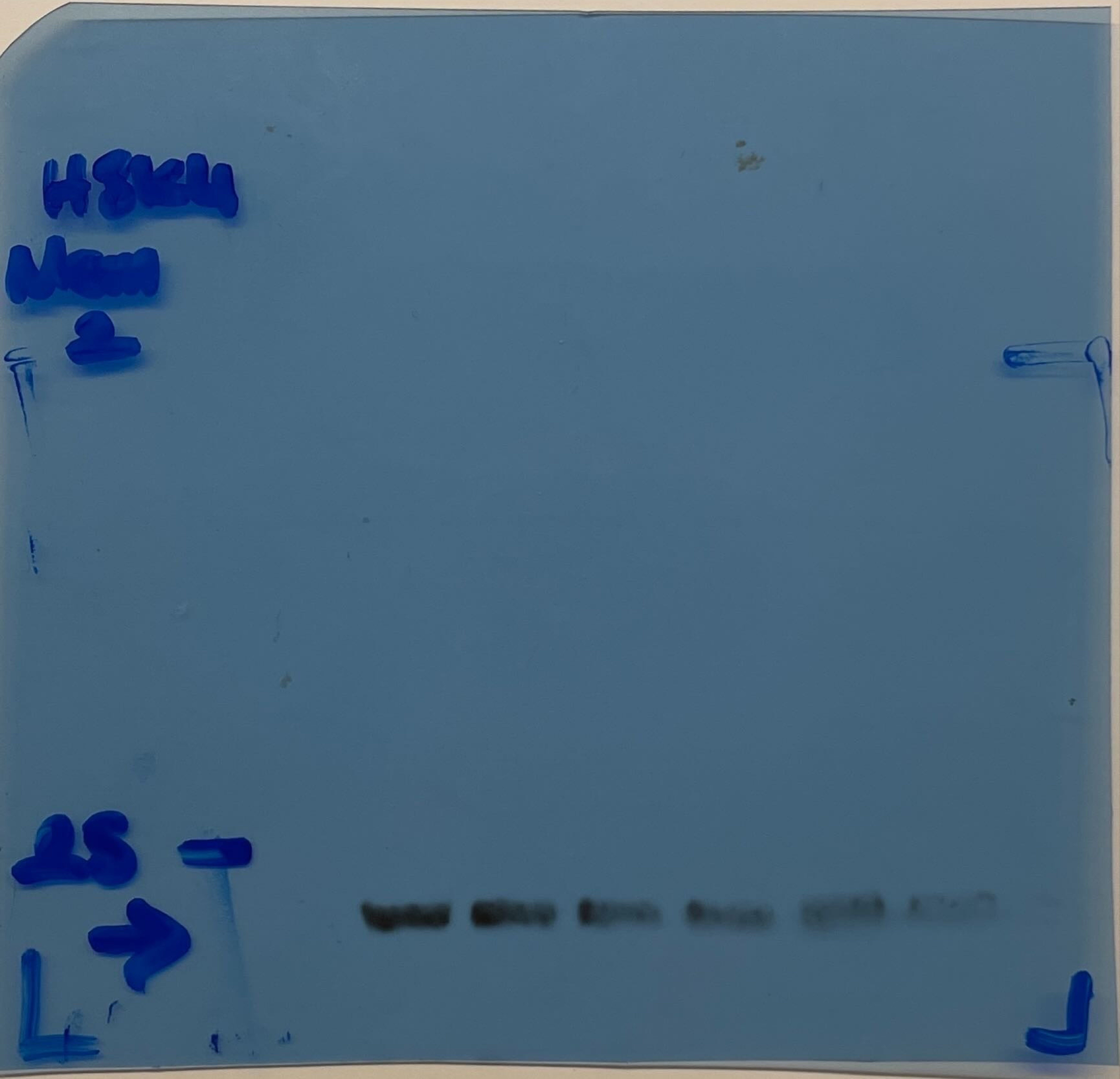
25

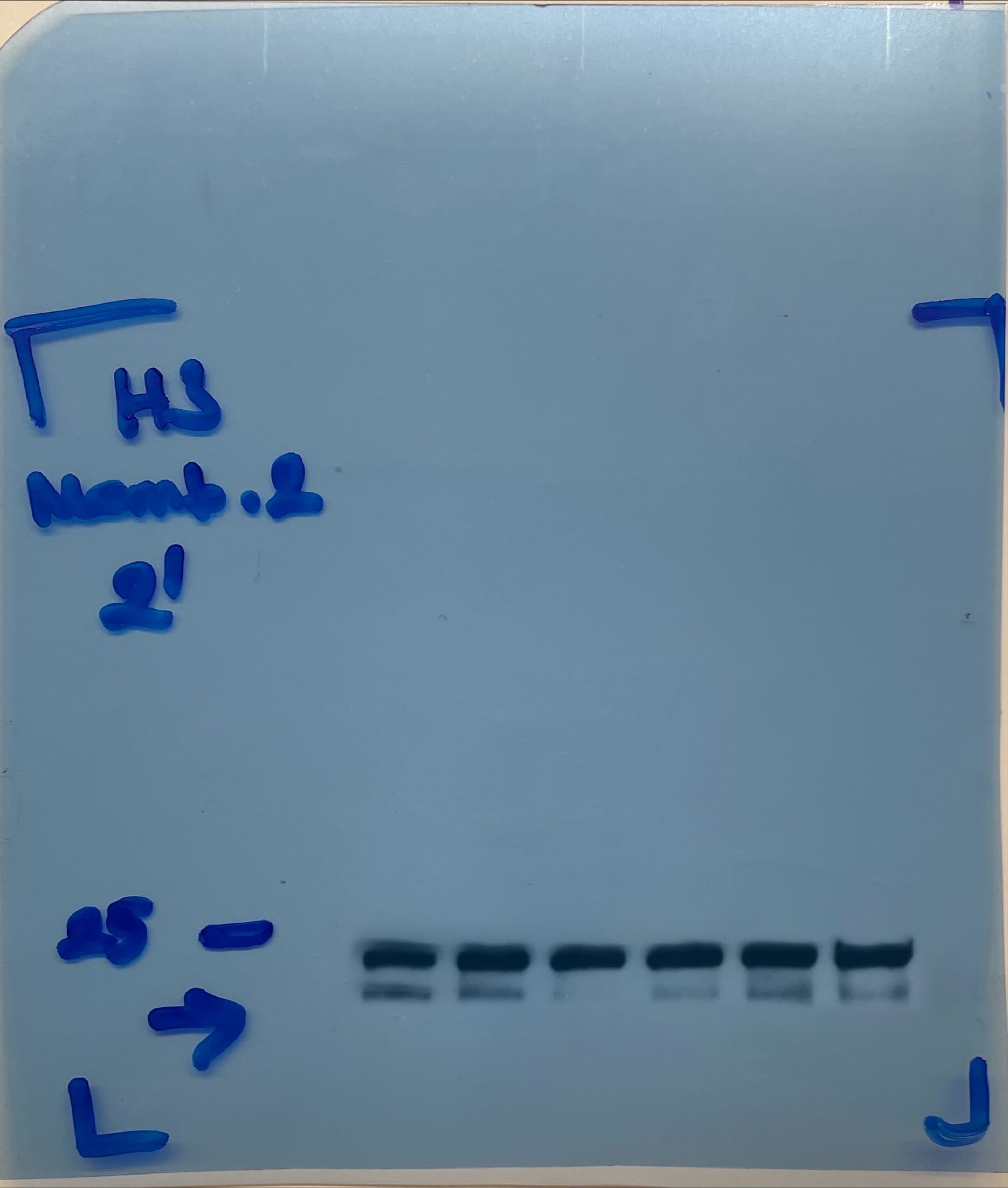
H3
2'
25

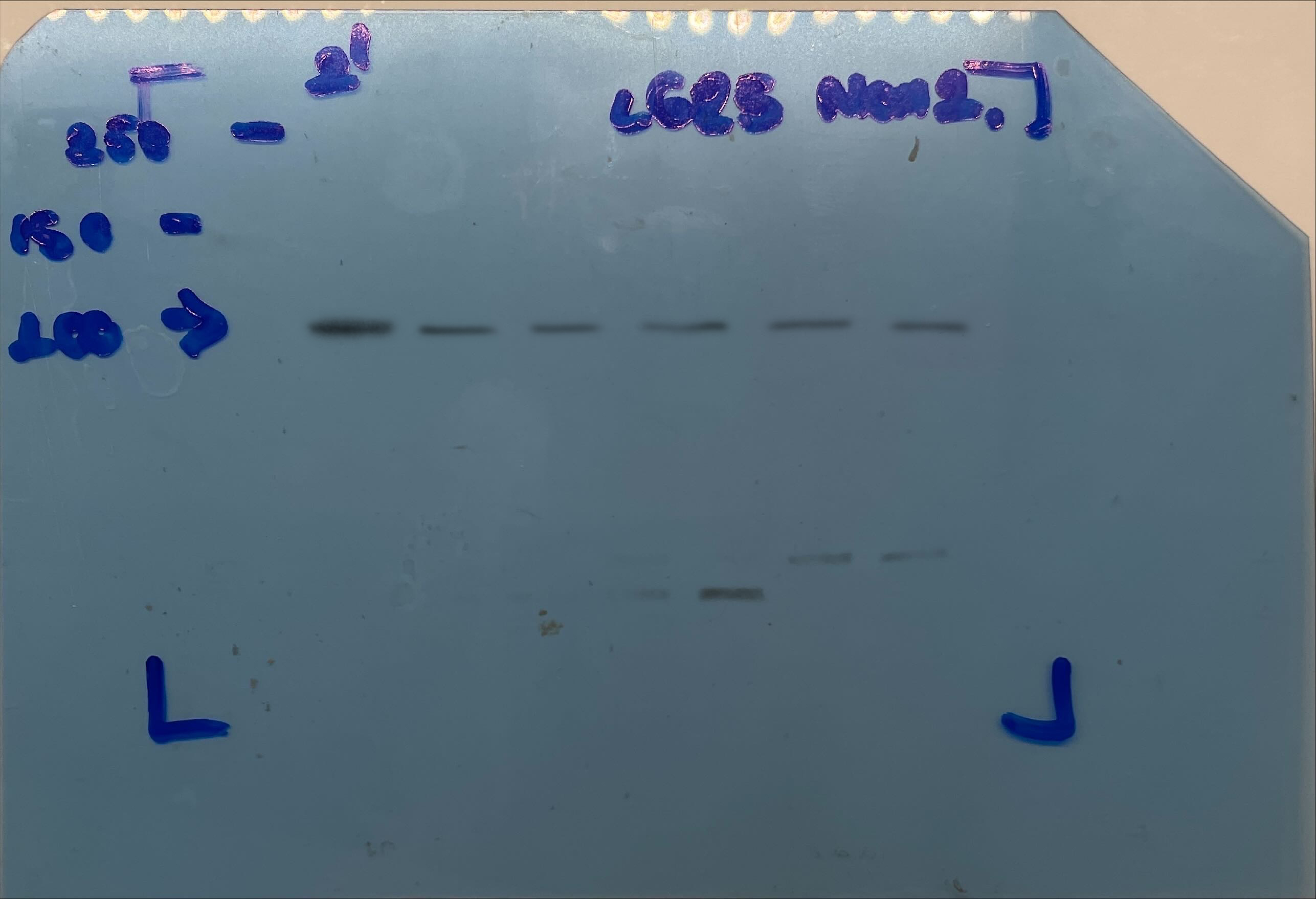
250
150
100

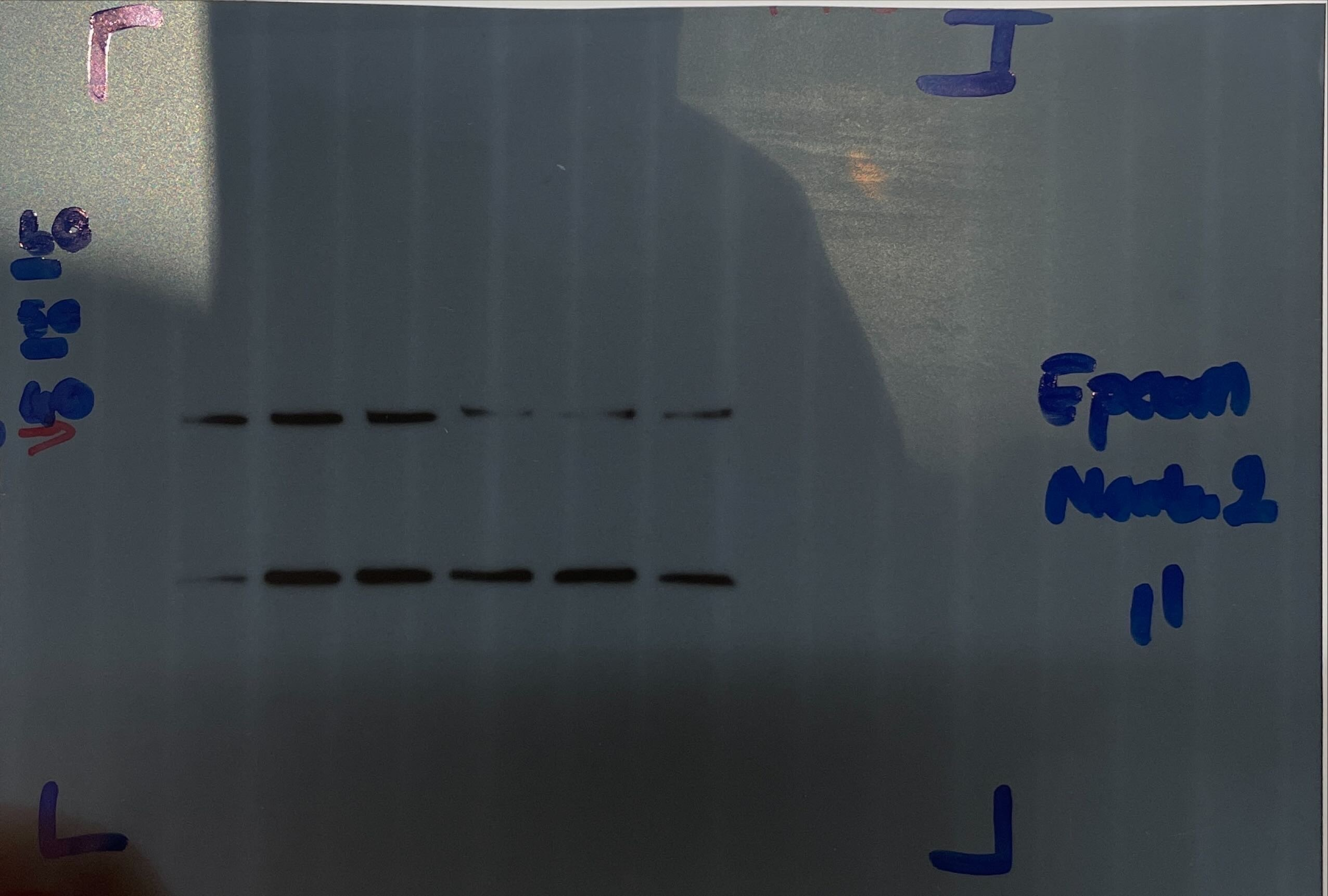
Epcam

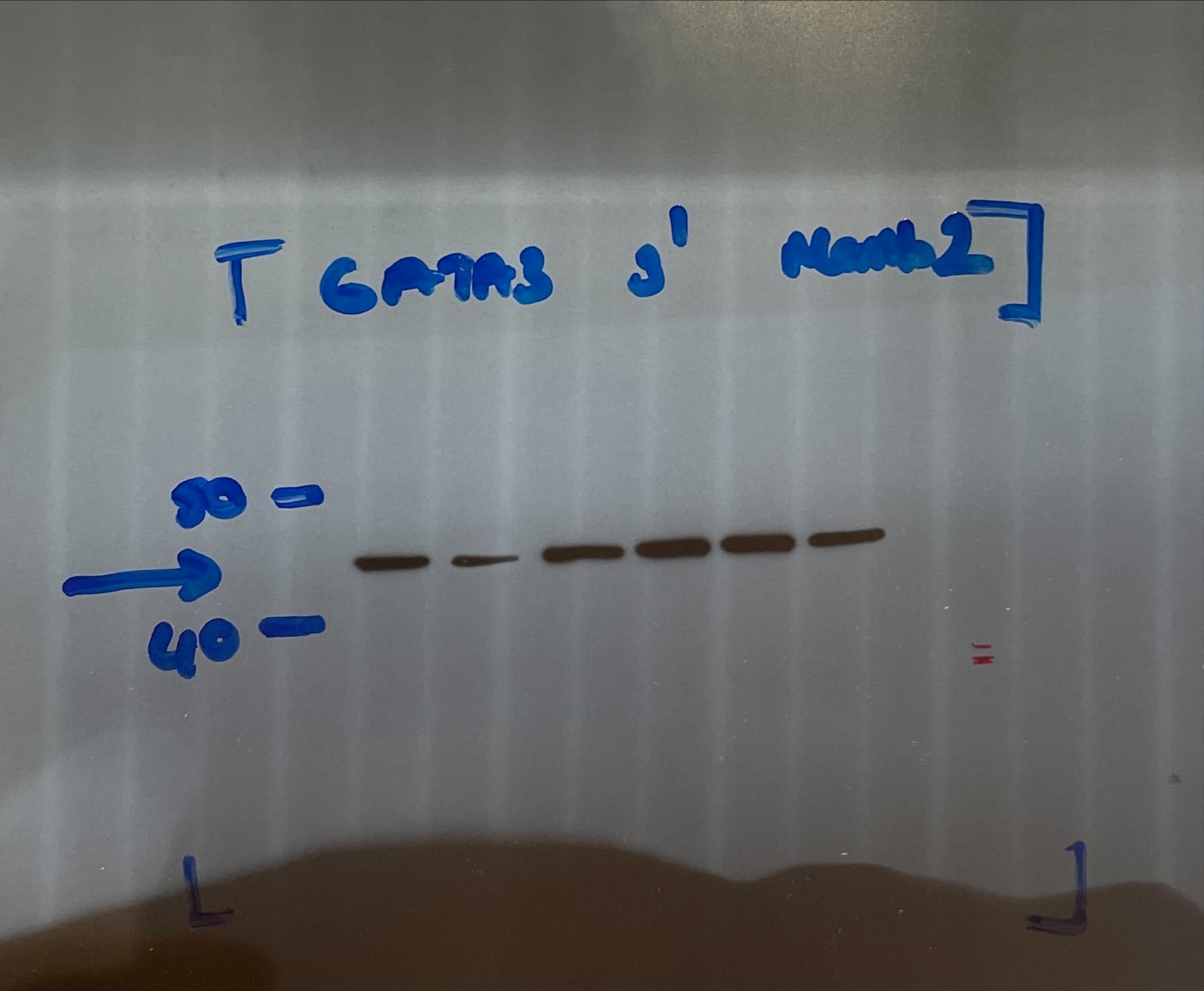
GATA3
50
40

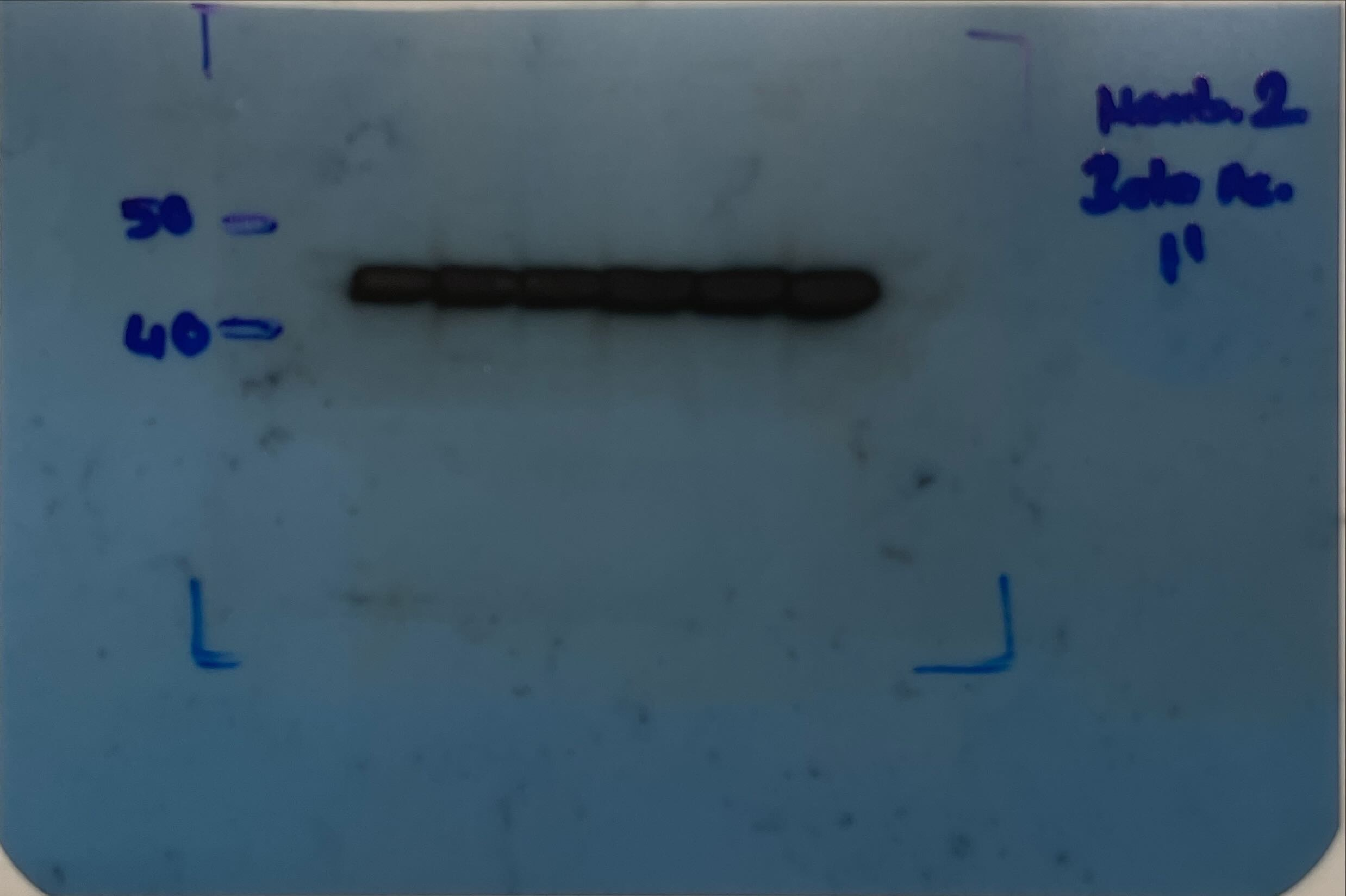
50
40

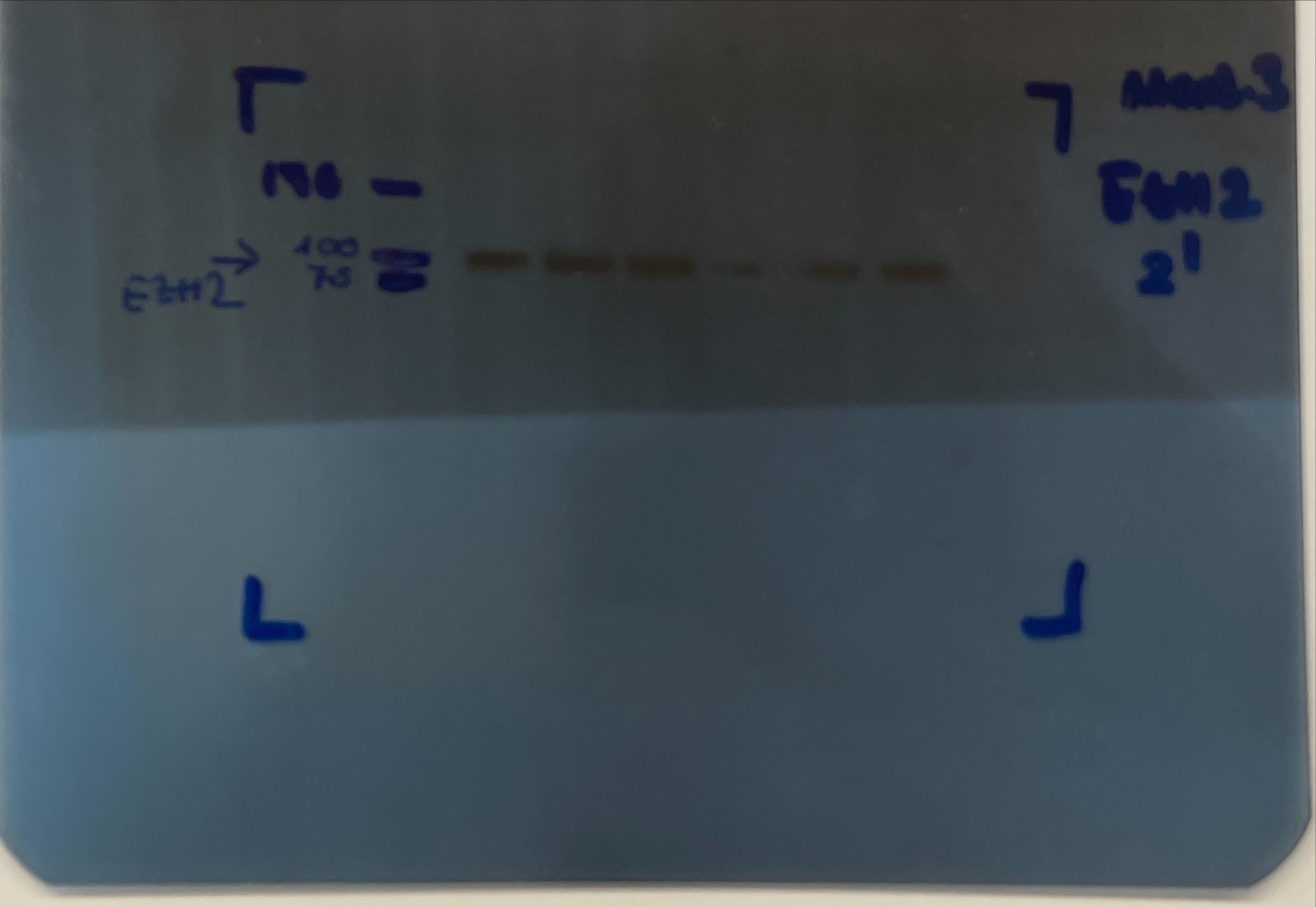
EZH2
100
75
EZH2

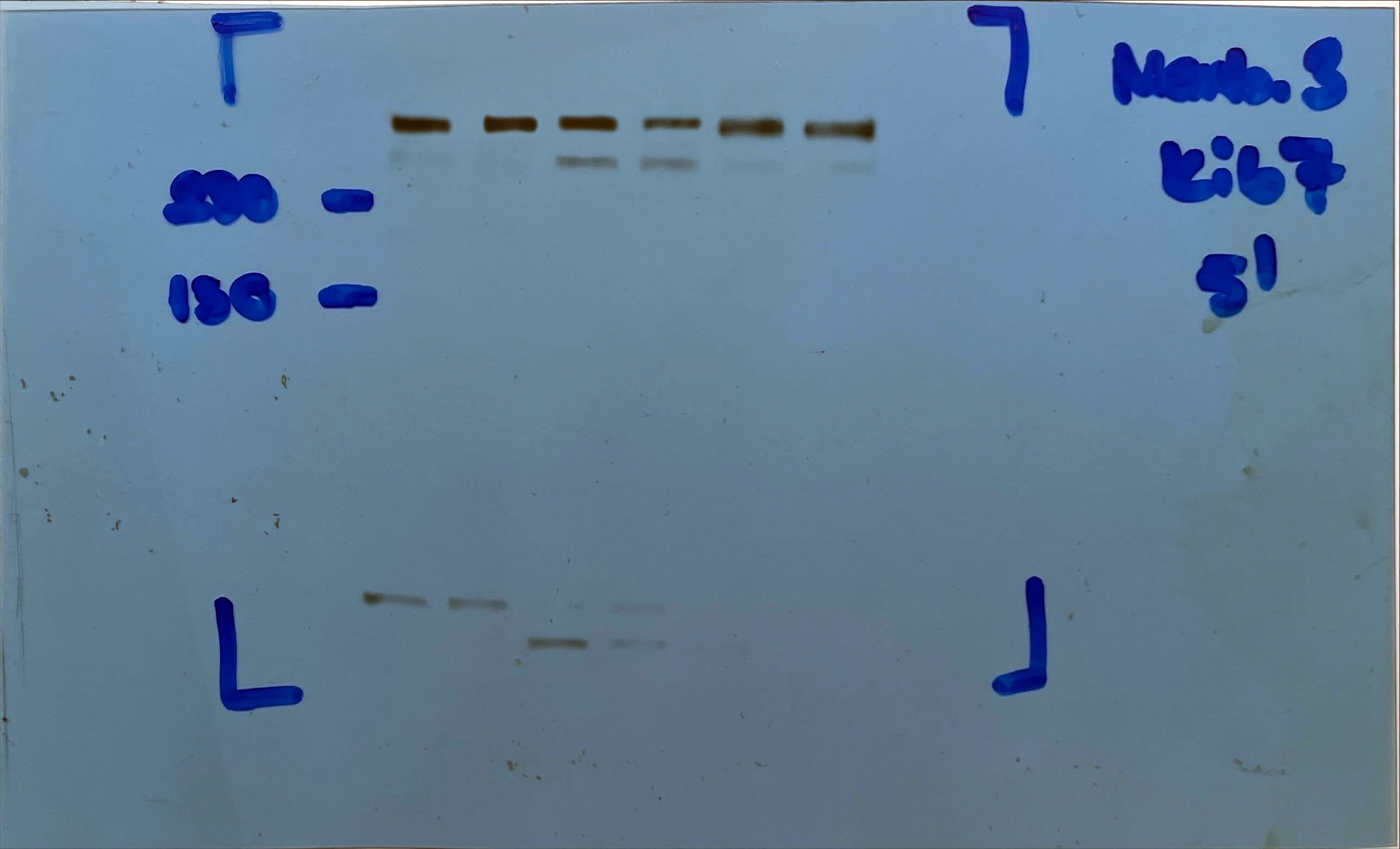
250
150
Ki67
5'

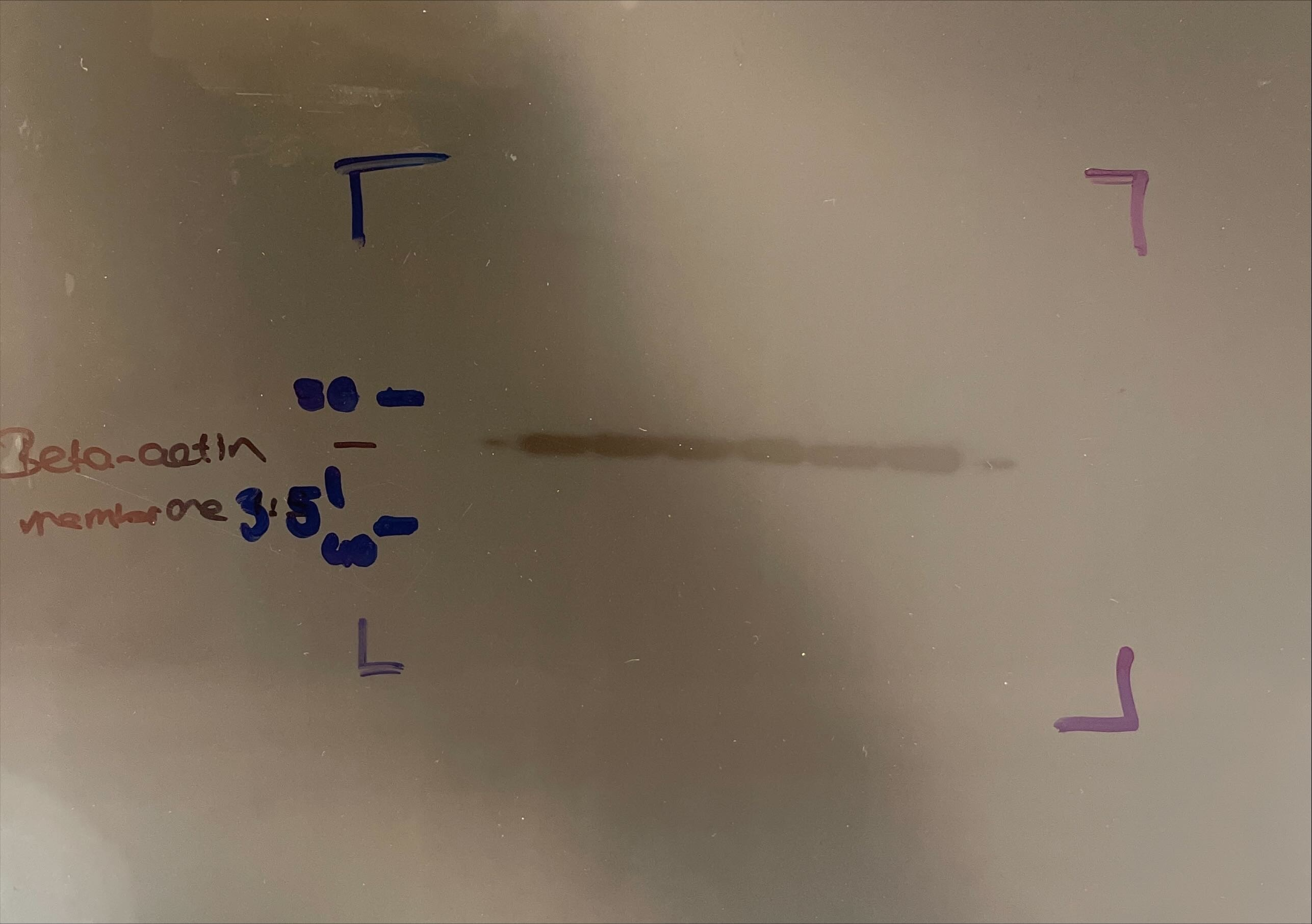
50
Beta-actin
40

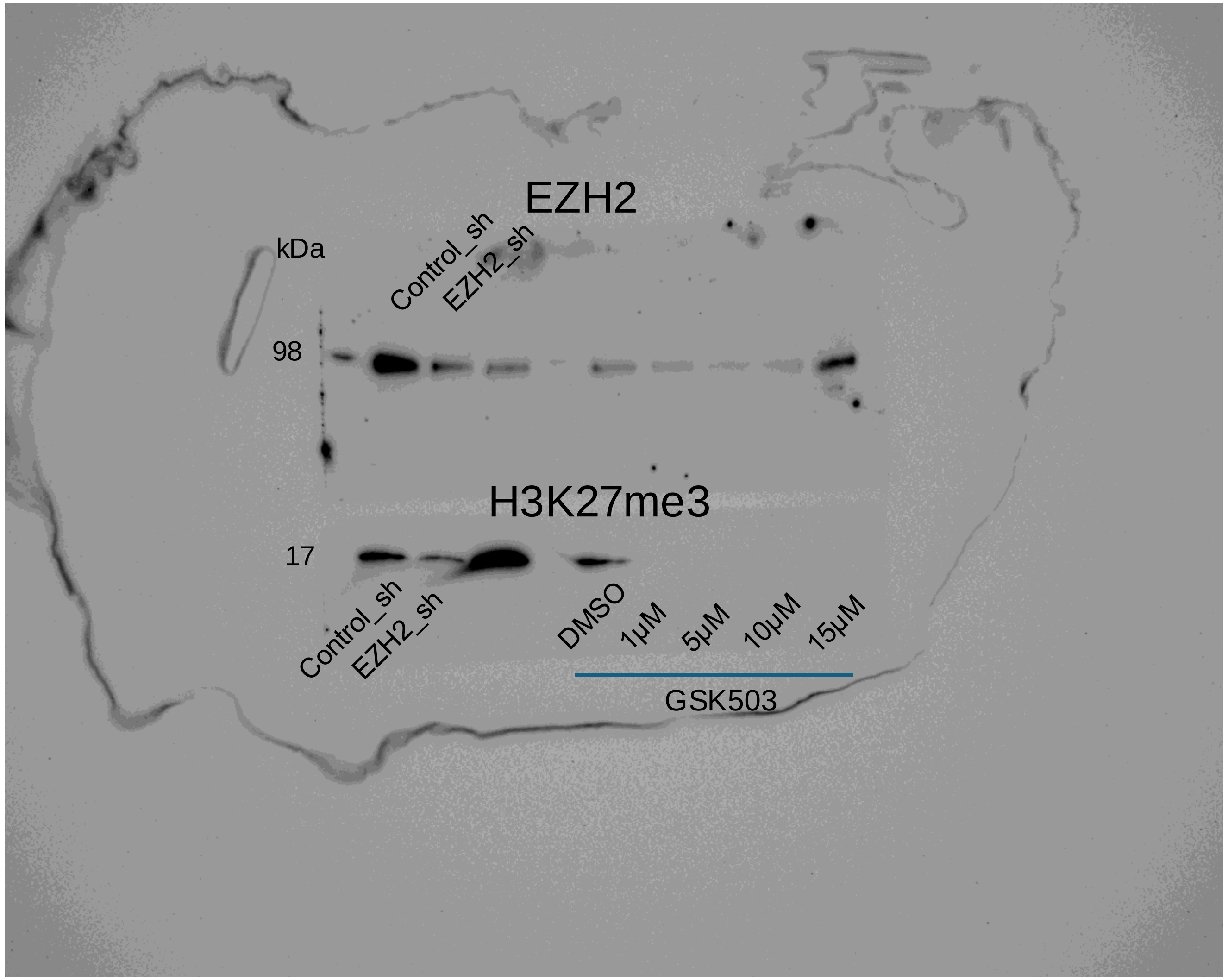
EZH2
kDa
Control_sh
EZH2_sh
98
H3K27me3
17
Control_sh
EZH2_sh
DMSO
1μM
5μM
10μM
15μM
GSK503

Histone H3

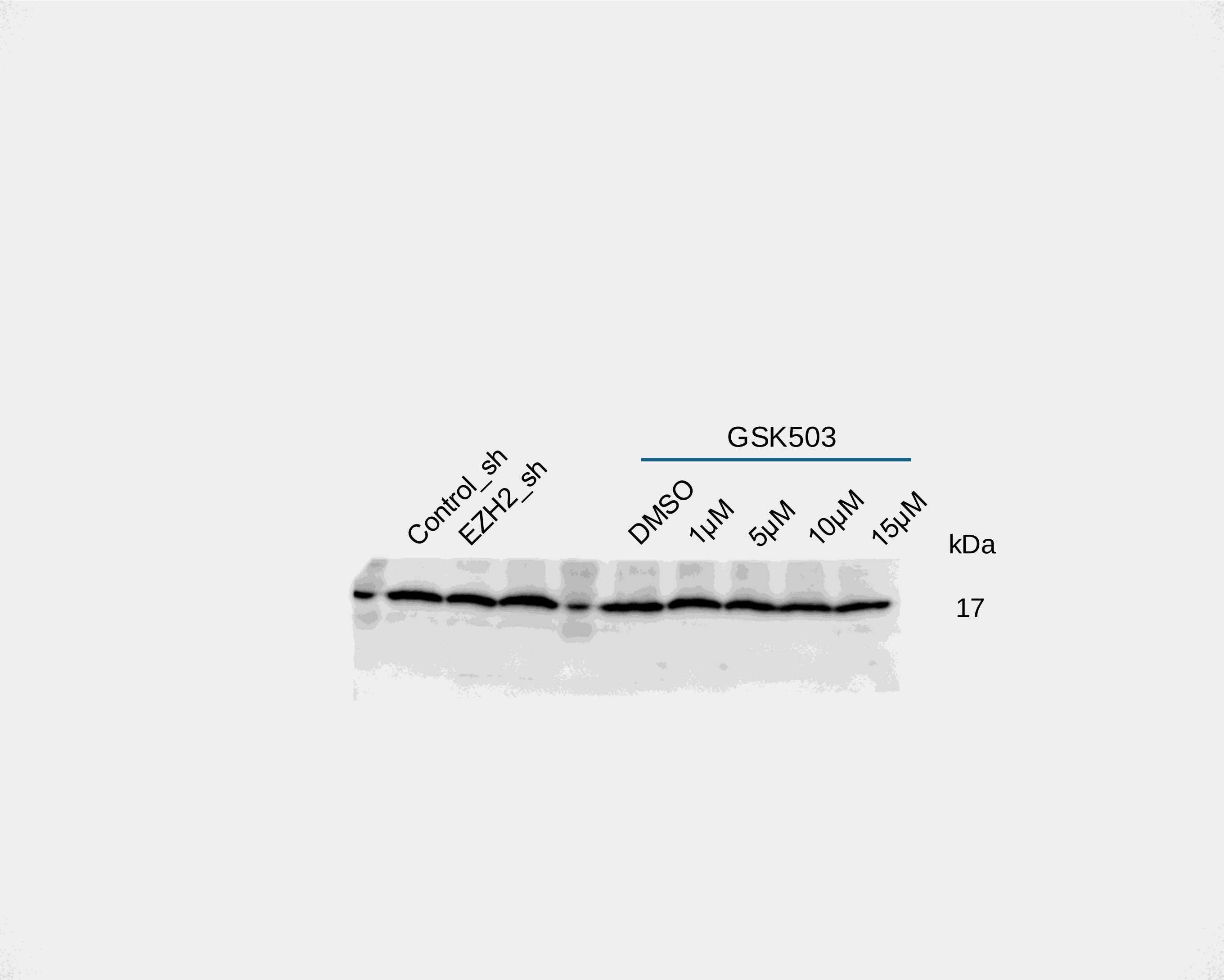

Beta-Actin_Supplemental WBs

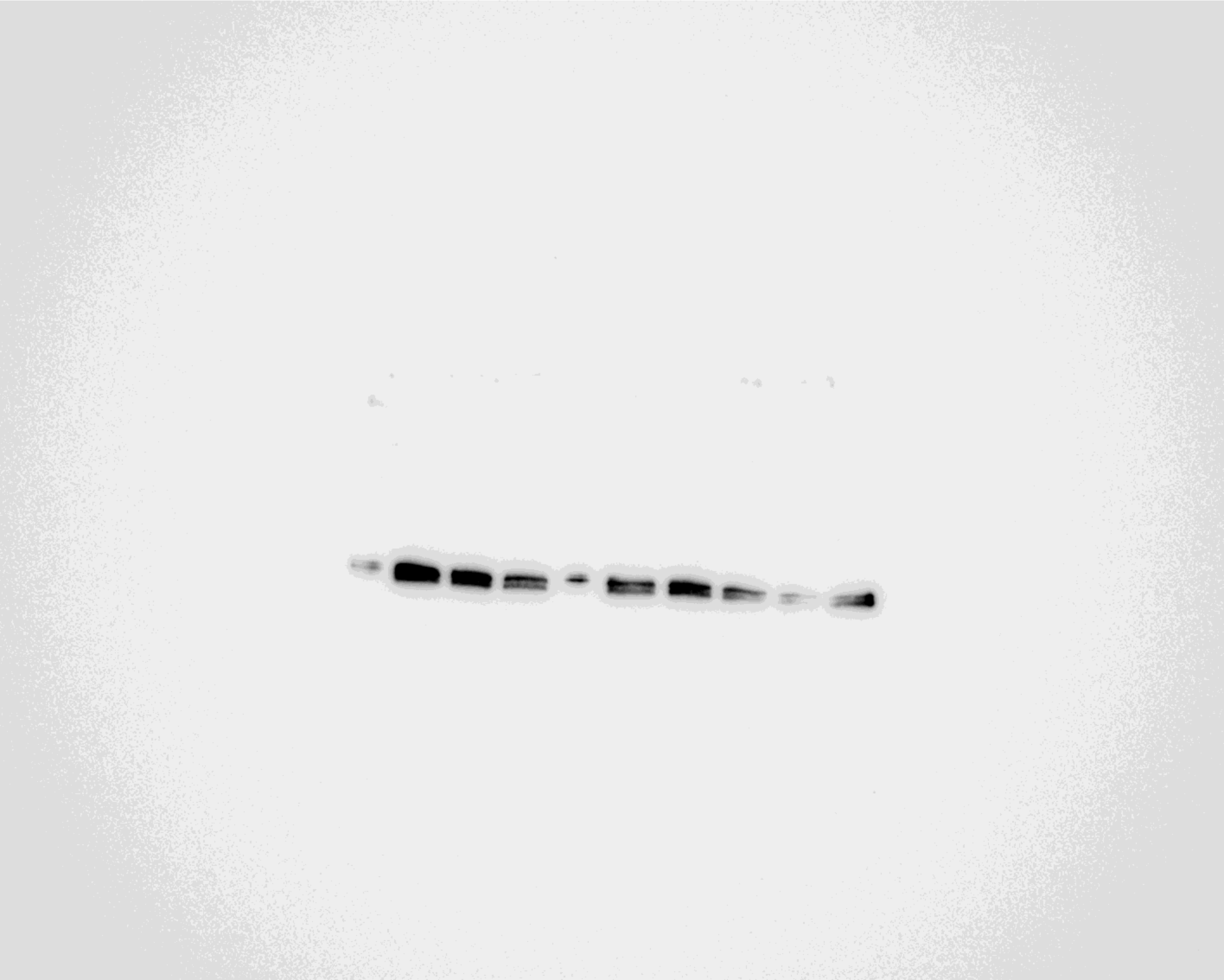

Supplement: Unedited blot and gel images [file jciinsight-10-177545-s283.pdf]
